# Pharmacophore-based virtual screening approaches to identify novel molecular candidates against EGFR through comprehensive computational approaches and *in-vitro* studies

**DOI:** 10.3389/fphar.2022.1027890

**Published:** 2022-11-15

**Authors:** F A Dain Md Opo, Mohammed Moulay, Ali Zari, Afnan Alqaderi, Saleh Alkarim, Talal Zari, Mohiuddin Ahmed Bhuiyan, Maged Mostafa Mahmoud, Fadwa Aljoud, Mohd Suhail, Sherif Edris, Wafaa S. Ramadan, Mohammad Amjad Kamal, Saïd Nemmiche, Foysal Ahammad

**Affiliations:** ^1^ Department of Biological Science, Faculty of Sciences, King Abdulaziz University, Jeddah, Saudi Arabia; ^2^ Embryonic Stem Cell Research Unit, King Fahd Medical Research Center, King Abdulaziz University, Jeddah, Saudi Arabia; ^3^ Embryonic and Cancer Stem Cell Research Group, King Fahd Medical Research Center, King Abdulaziz University, Jeddah, Saudi Arabia; ^4^ King Fahd Medical Research Center, King Abdulaziz University, Jeddah, Saudi Arabia; ^5^ Department of Biology, Abdelhamid ibn Badis University, Mostaganem, Algeria; ^6^ Department of Pharmacy, University of Asia Pacific, Dhaka, Bangladesh; ^7^ Molecular Genetics and Enzymology Department, Human Genetics and Genome Research Institute, National Research Centre, Cairo, Egypt; ^8^ Regenerative Medicine Unit, King Fahd Medical Research Center, King Abdulaziz University, Jeddah, Saudi Arabia; ^9^ Department of Medical Laboratory Sciences, Faculty of Applied Medical Sciences, King Abdulaziz University, Jeddah, Saudi Arabia; ^10^ Princess Al-Jawhara Al-Brahim Centre of Excellence in Research of Hereditary Disorders (PACER-HD), Faculty of Medicine, King Abdulaziz University, Jeddah, Saudi Arabia; ^11^ Department of Anatomy, Faculty of Medicine, King Abdulaziz University, Jeddah, Saudi Arabia; ^12^ Department of Pharmacy, Faculty of Allied Health Sciences, Daffodil International University, Dhaka, Bangladesh; ^13^ Division of Biological and Biomedical Sciences (BBS), College of Health and Life Sciences (CHLS), Hamad Bin Khalifa University (HBKU), Doha, Qatar

**Keywords:** EGFR, side effects, cell toxicity, *in-vitro*, inhibitors, gefitinib

## Abstract

Alterations to the EGFR (epidermal growth factor receptor) gene, which primarily occur in the axon 18–21 position, have been linked to a variety of cancers, including ovarian, breast, colon, and lung cancer. The use of TK inhibitors (gefitinib, erlotinib, lapatinib, and afatinib) and monoclonal antibodies (cetuximab, panitumumab, and matuzumab) in the treatment of advanced-stage cancer is very common. These drugs are becoming less effective in EGFR targeted cancer treatment and developing resistance to cancer cell eradication, which sometimes necessitates stopping treatment due to the side effects. One *in silico* study has been conducted to identify EGFR antagonists using other compounds, databases without providing the toxicity profile, comparative analyses, or morphological cell death pattern. The goal of our study was to identify potential lead compounds, and we identified seven compounds based on the docking score and four compounds that were chosen for our study, utilizing toxicity analysis. Molecular docking, virtual screening, dynamic simulation, and *in-vitro* screening indicated that these compounds’ effects were superior to those of already marketed medication (gefitinib). The four compounds obtained, ZINC96937394, ZINC14611940, ZINC103239230, and ZINC96933670, demonstrated improved binding affinity (−9.9 kcal/mol, −9.6 kcal/mol, −9.5 kcal/mol, and −9.2 kcal/mol, respectively), interaction stability, and a lower toxicity profile. *In silico* toxicity analysis showed that our compounds have a lower toxicity profile and a higher LD_50_ value. At the same time, a selected compound, i.e., ZINC103239230, was revealed to attach to a particular active site and bind more tightly to the protein, as well as show better *in-vitro r*esults when compared to our selected gefitinib medication. MTT assay, gene expression analysis (BAX, BCL-2, and β-catenin), apoptosis analysis, TEM, cell cycle assay, ELISA, and cell migration assays were conducted to perform the cell death analysis of lung cancer and breast cancer, compared to the marketed product. The MTT assay exhibited 80% cell death for 75 µM and 100µM; however, flow cytometry analysis with the IC_50_ value demonstrated that the selected compound induced higher apoptosis in MCF-7 (30.8%) than in A549.

## 1 Introduction

The epidermal growth factor receptor (EGFR) works through attachment with the ligand and maintains cell growth through autophosphorylation by activating different signal transduction. The EGFR family consists of four different subgroups: erbB1, erbB2, erbB3, and erbB4 (Bethune et al., 2010). Mutation in this group of genes shows the development of several cancers in humans, such as lung cancer, colorectal cancer, breast cancer, liver cancer, pancreatic cancer, and ovarian cancer (Krasinskas 2011; Rahman et al., 2022). The most common cancer is non-small-cell lung cancer caused by mutation of EGFR in exon 19 and L858R positions (Yan et al., 2020). Mutation was also found at positions E18, E19, E20, and E21 among male and female cancer patients in the study conducted on 1,020 patients from 2010 to 2016 (Yoon et al., 2020). Currently, tyrosine kinase (TK) inhibitors are available in the market as first line of drugs such as gefitinib and erlotinib to treat non-small-cell lung cancer (NSCLC), but the tumors are going to be resistant against these drugs due to secondary mutation in T790M. Second-generation drugs have already shown negative results in clinical trial against the T790M mutation, but third-generation drugs such as WZ4002, rociletinib, and osimertinib are a better choice against T790M mutated lung adenocarcinoma. A study showed that in colorectal cancer, EGFR protein overexpressed from 25% to 82% and one available drug was in the phase II clinical stage to treat patient (Spano et al., 2005). A study conducted among American, Chinese, and Korean triple negative breast cancer patients found EGFR mutation, which has been identified in exon 21 for Americans and exons 19 and 21 for both Chinese and Korean populations (Kim et al., 2017). EGFR mutation in breast cancer patients is increasing and identified using the Sanger sequencing process. Mutation in T790M in Norwegian breast cancer patients has also been found by the real time-polymerase chain reaction (RT-PCR) process although they were not previously taking any cancer medicine. Currently available TK inhibitors (gefitinib and erlotinib) for breast cancer treatment did not show efficient treatment in clinical trials (Bemanian et al., 2015; Nishikawa et al., 2018). Gefitinib is known to be a well-tolerated drug, which showed synergistic effects in combination with tamoxifen in breast cancer treatment (Normanno et al., 2006). In lung cancer treatment, the initial clinical response is satisfactory but eventually resistance is developed due the mutation in EGFR (T790M) (Pao et al., 2005), MET gene amplification, and the activation of NF- κB and TGF-β (Engelman et al., 2007; Bivona et al., 2011; Huang et al., 2012). MET is a tyrosine kinase receptor that contributes to produce hepatocyte and mutation of this gene causes to develop liver carcinoma. For advanced hepatocellular cellular carcinoma, erlotinib is a good choice for treatment, but it is growing to be resistant in cases of hepatocellular carcinoma (HCC) and breast cancer treatment (Bartholomeusz et al., 2011; Rahman et al., 2021). Therefore, the development of alternative EGFR inhibitors is necessary to overcome drug resistance and sensitivity for EGFR-based targeted treatment in the lung, breast, liver, and other organs (Shi et al., 2022). Our selected protein with its attached ligand showed an increased safety profile in the *in silcio* study than other first- and second-generation drugs and was approved by the Food and Drug Administration (FDA) to treat metastatic EGFR containing T790M mutation. The toxicity profile evaluation of our selected four compounds indicated that they had more binding energy and better toxicity results compared to the control ligand and gefitinib.

The method for developing therapeutics against a specific target with computer software is known as computer-aided drug design (CADD), also known as *in silico* drug design. In comparison to traditional drug design, modern CADD techniques such as pharmacophore modeling, molecular docking, ADMET (absorption, distribution, metabolism, excretion, and toxicity), and molecular dynamics (MD) simulation can produce hits for lead compounds more quickly (Valasani et al., 2014; Macalino et al., 2015; Bommu et al., 2018; Opo et al., 2021). In traditional drug development, collected blood samples from clinical trials or *in vivo* experiments can be used to examine the ADMET parameters (Beaumont et al., 2014). The CADD approach is a popular alternative method that makes it simpler and more effective to analyze the ADMET parameters. The methods can identify a wide range of organ toxicity and other aspects, suggesting the possibility of conflicting effects (Aljahdali et al., 2021). The second steps in drug development were also articulated by molecular biologists, who could likewise predict potential cell death at certain concentrations. Different ligands and structure-based computational studies were performed on different compounds with a lack of toxicity, simulation analysis, and *in-vitro* studies (Sangande et al., 2020). Utilizing *in silico* drug design, our work sought to identify additional possible lead compounds against the EGFR overexpressed protein and explore the possibility to overcome the T790M mutation. However, extensive animal-based experimental research studies are necessary to evaluate the efficacy of these potential lead compounds.

## 2 Materials and methods

### 2.1 Structure-based 3D-pharmacophore modeling

#### 2.1.1 Pharmacophore modeling

**FIGURE 1:**
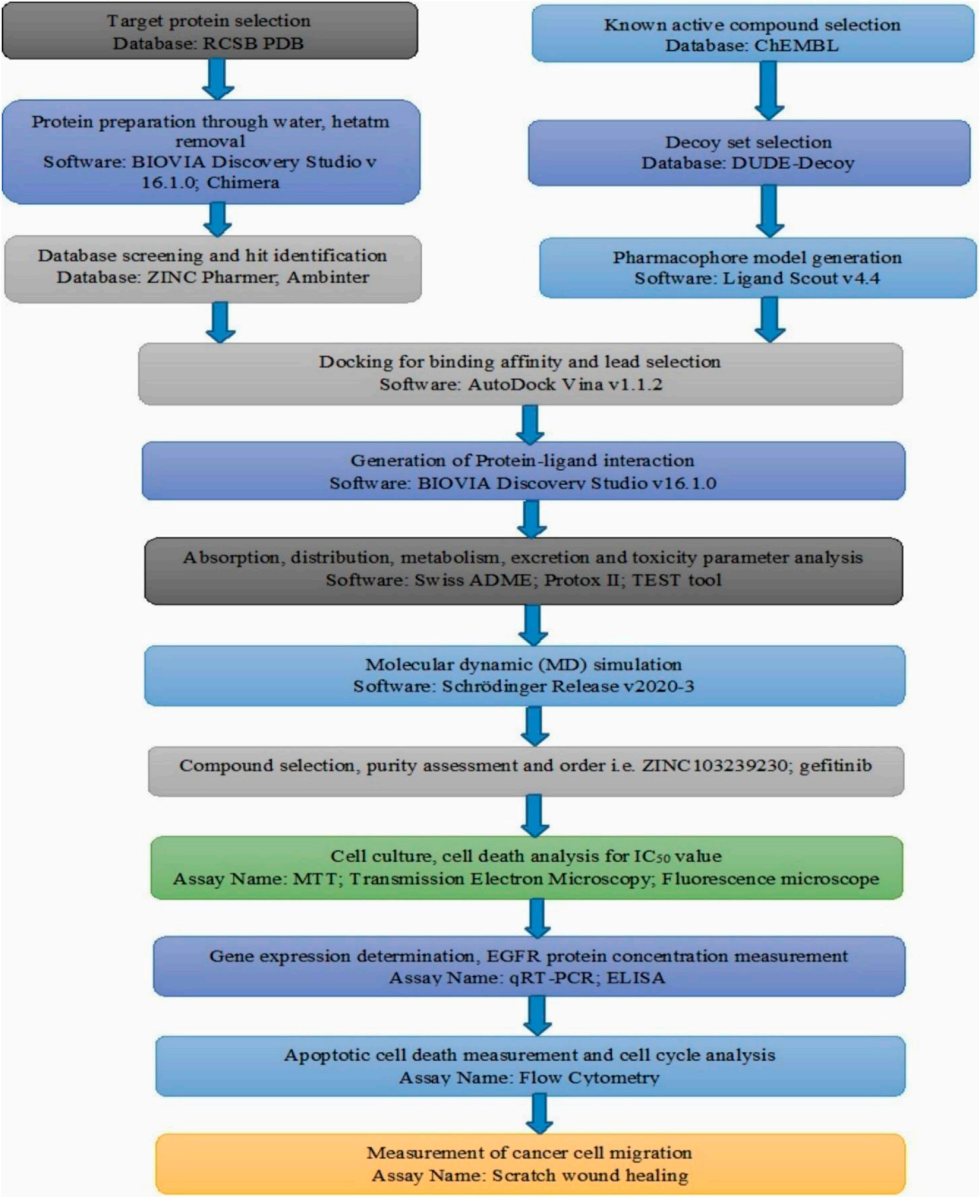
Overall flow chart of our conducted research based on the computer-aided drug design and *in-vitro* analysis.

The crystal structure of epidermal growth factor receptor protein (PDB ID: 6JXT) was obtained using the online RCSB protein data bank (Berman et al., 2000). Ligands attached to the selected protein were subjected to screening against a compound library to get similar lead compounds. The widely used ligand scout v4.4 advance tool was used to prepare structure-based pharmacophore model based on the cooperation of inhibitors (Wolber and Langer 2005). This software can be used for the deletion or addition of any features for better screening, including negative or positive ionization, hydrogen bond acceptor, hydrogen bond donor, charge transfer, and the addition of hydrophobic or hydrophilic regions. The overall methodology for our research work has been provided by the flow chart (Figure 1).

#### 2.1.2 Pharmacophore model validation

In the structure-based pharmacophore preparation, the interaction pattern of our ligand and its target molecule can be obtained through ligand–target complexes. A set of chemical compounds known as antagonists were identified based on the ChEMBL database (Davies et al., 2015) and through several literature searches had an IC_50_ value. The directory of useful decoys enhanced (DUD-E) database was used to identify the decoy compounds after getting the smile file from chemical databases and shifted to the ligand scout v4.4 software tool for making an “idb” file for further analysis (Mysinger et al., 2012). We evaluated the accuracy of our model based on early enrichment factor (EF). The early enrichment factor indicated the availability of active molecules from the decoy set analysis. Receiver operativing curve (ROC) analysis showed how to get both active and inactive compounds at a time; although inactive compounds might have a similar structure, they are not generated in ROC preparation.

### 2.2 Virtual screening based on the pharmacophore model

#### 2.2.1 Dataset generation

A freely assessable database, the ZINC database (https://zinc15.docking.org/substances/home/), was used to identify structurally novel and active molecules. It is a chemical database having small molecules to large molecules, and the desired compounds can be searched based on the chemical structure, compound names with their targets, chemical ID, and smile file also. In our study, compounds were selected based on maximum matching features with our desired pharmacophore features and for the possibility of easy interaction with our selected protein (Irwin et al., 2012). The compounds with a molecular weight of less than 500 kDa and an RMSD value of less than or equal to 1 were obtained from searching the natural products, natural derivatives, and purchasable compounds of the ZINCPharmer database by utilizing the selected ligand pharmacophore characteristics generated by the ligand scout v4.4 advance tool. The maximum number of compounds is chosen based on the highest number of features directly related to the query pharmacophore. The identified compounds were further subjected to analyze several features on the basis of Lipinski’s rule of five. Selected molecules were saved and proceeded for further validation.

#### 2.2.2 Virtual screening

Structurally, similar lead compounds can be identified from the diverse chemical database. These compounds can easily be obtained from available commercial sources in the market for further analysis (Pokhrel et al., 2021). A previously generated dataset was subjected to screening based on using our selected pharmacophore properties. Ligand Scout v4.4 advance software was explored to generate the “idb” file using load screening database features and submit it to the pharmacophore-data-based bank. Hit was found as a scoring function. We selected a high fit value as it indicates good matches, and compounds were subjected for more authentication.

### 2.3 Docking-based virtual screening

#### 2.3.1 Structural analysis of ligands and proteins

The crystal structure of protein was downloaded from the protein data bank. EGFR protein attached with two ligands YY3 and Cl^−^ was selected from the RCSB protein data bank for docking studies based on several parameters, i.e., source, experimental methods, refinement resolution, release date, IC_50_ value, and toxicity results of the attached ligand. The resolution was 2.31 Å, with other parameters including R-value free: 0.239, R-value work: 0.216, and R-value observed: 0.218. Hetatm and other ligands were deleted using the BIOVIA Discovery Studio v16.1.0. Auto dock tools were used to add Kollaman charges, and Gasteiger and PyRx v0.9 were used to minimize the energy for better docking (Dallakyan and Olson 2015).

#### 2.3.2 Binding site and grid selection

The drug’s efficiency depends on the proper binding to the target area. Improper binding may cause drugs to produce less efficacy, side effects, or toxic effects (Brylinski 2017). The active site of a protein was identified based on the research article analysis and using CASTp software v3.0. Binding affinities depend on the number of H-bond donors or acceptors, hydrophobic or hydrophilic interaction, positive and negative ionization features, and the presence of chelation features (Meng et al., 2011). We selected a binding site without any assumption for better binding or any new binding site, known as blind binding. Grid preparation was completed through the selection of functional parts of the protein by PyRx software v0.9 with the dimensions (X: 26.69, Y: 34.28, and Z: 31.14) (Dallakyan and Olson 2015).

#### 2.3.3 Molecular docking

Currently, molecular docking is being widely used to study the interaction between proteins and ligands (Dain Md Opo et al., 2022). Our desired protein was found by analyzing the RCSB protein data bank (PDB ID: 6JXT). The docking areas of proteins and compounds were identified through PyRx software v0.9 (Dallakyan and Olson 2015). The water molecules were removed by the BIOVA Discovery Studio Visualizer Tool (v16.1.0), except the interacting one with the inhibitor, and the chain was selected from our chosen protein. In our experiment, Autodock Vina (Version 1.1.2) was used to examine interactions between ligands and proteins (Trott and Olson 2010). Protein energy was minimized before proceeding to docking, and further selection was completed based on the maximum negative value (binding energy).

### 2.4 Pharmacokinetic parameter (ADME) and toxicity evaluation

#### 2.4.1 Absorption, distribution, metabolism, and excretion tests

ADME profile investigation is an important parameter to get the possible effect of a drug on the body through administration. Its pharmacokinetic profile and pharmacodynamic activities can be evaluated (Nisha et al., 2016; Dain Md Opo et al., 2022). The ADME profile analysis is necessary to overcome the failure of drugs during the clinical trial phase. We used online Swiss ADME (http://www.swissadme.ch/index.php). The drug likeliness property and medicinal chemistry related characteristics were also obtained from the server (Yadav et al., 2017; Opo et al., 2021).

#### 2.4.2 Toxicity test

Early toxicity identification is a crucial component of drug research and development in order to prevent late-stage drug development failure (Hughes et al., 2011). The Toxicity Estimation Software Tool (TEST) v4.2.1 and Protox II (http://tox.charite.de/protox_II) were used to calculate the toxicity level for most of our selected compounds, and it worked by scanning the quantitative structure-activity relationship (QSAR) methodologies (Banerjee et al., 2018). QSAR works on the physical features and the structural behavior of the chemicals (Martin et al., 2017). We evaluated the compounds’ mutagenicity, carcinogenicity, cardiotoxicity, oral toxicity, hepatotoxicity, and respiratory toxicity. Several drug-related properties such as 48-h D. magna LC_50_, 48-h T. pyriformis IGC_50_, lethal dose 50, bioaccumulation factor, developmental toxicity, and mutagenicity have been calculated.

### 2.5 Molecular dynamic simulation

The protein binding interaction of our selected molecules and ligands containing EGFR protein were evaluated by the working Desmond module of Schrödinger Release 2020-3 (Academic version) based on the Linux system to confirm the structural integrity of the protein complex (Bowers et al., 2006; Dain Md Opo et al., 2022). For better binding complex identification, we carried out 100ns of simulations using the optimized potentials for liquid simulations (OPLS) 2005 force field, maintaining pH 7.4. The chosen protein and selected complex were first solvated with water molecules, and boundaries were provided to the complex with an orthorhombic box. Na^+^ and Cl^−^ charges were adjoined to nullify charges, keeping salt concentration at 0.15M. The simulation was completed at 1.01325 bar pressure with a 300K constant temperature through the mainlining recording interval of 5ps. A protein root mean square deviation (RMSD) value was calculated and determined based on the selected atom. The stability of the ligand protein complex was identified by RMSD, RMSF, and SSE values. Ligand interactions with different atoms also determined each trajectory frame. From the trajectory file, the radius of gyration analysis indicated the structural compression during the 100ns simulation.

### 2.6 Compound purchased and stock preparation

Briefly, 5 mg of the compound was obtained from the S Molecules (Q-11505, Supplementary Table S1), and the tyrosine kinase inhibitor, gefitinib, was obtained from a renowned pharmaceutical company of Bangladesh. Tablets were purchased from a local pharmacy outlet of Dhaka. The tablets were crushed in the autoclaved mortar and pestle. As the tablet contained several excipients, we calculated the total active amount necessary from the whole tablet by measuring the total tablet weight. A 10 mM stock was prepared for both the compounds.

### 2.7 *In-vitro* studies with A549 and MCF-7

#### 2.7.1 Cell culture and subculture

Breast cancer (MCF-7) and lung cancer (A549) cells were collected and thawed using a 37 C water bath. The cells were transferred to the 15 ml falcon tube for centrifugation for 4 min at 1200rpm. The pellets were collected by discarding the supernatant and resuspended with 2 ml of DMEM media. A small number of cells were collected and transferred to the T25 cm^2^ cell culture flask (SPL, South Korea). When the cell number reached the optimum density, they were then trypsinized, centrifuged at 1200 rpm, and the flask was kept in a CO_2_ incubator, maintaining a specific temperature (37 C).

#### 2.7.2 Cell toxicity assay

It is the most popular assay to determine the cell inhibition rate in the presence of an inhibitor. A specific number (1 × 10^4^) of cancer (MCF-7, A549) cells were counted and seeded in a 96-well plate and the plate was transferred to a CO_2_ incubator for 24 h. The cells were mixed with the our selected compounds and marketed drugs with the same concentrations for 48 h. The old culture medium was removed, and a fresh culture medium containing MTT chemicals was added. The plates were incubated until a purple color appeared, and the absorbance was determined by a microplate (ELISA) reader at 570 nm wavelength (Rahman et al., 2021).

#### 2.7.3 Cell morphological change analysis

Cancer cells (MCF-7 and A549) were plated at 5 × 10^5^ in numbers in the 25 cm^2^ flask and incubated for 24 h for their attachment. After the optimal growth cells were treated by the several IC_50_ concentrations of marketed drugs and our selected antagonist, the flasks were kept in the CO_2_ incubator for a specific time period, and cell morphology, cell proliferation, and cell death were evaluated. Photos were taken by a fluorescence microscope at different (×4, ×10) magnifications. Untreated cell lines were considered the control.

#### 2.7.4 Transmission electron microscopy analysis

All cancer (MCF-7 and A549) cells were used to find the morphological changes and cell death based on the drug concentration based on IC_50_. Previously cultured cancer cells were treated using the selected antagonist and kept in incubation for 48 h in the incubator at 37 C, whereas untreated cancer cells were used as controls. The cells were attached to the carbon-coated grid through a tiny drop for 15 min. The cells containing grids were air-dried before being analyzed by TEM.

#### 2.7.5 Apoptosis assay by flow cytometry

Previously counted (1×10^5^) fixed numbers of cancer cells (MCF-7 and A549) were plated in a flask of 25 cm^2^ and incubated for 24 h in the CO_2_ incubator at 37 C. After 24 h of incubation, cancer cells were treated by gefitinib and our selected compound with IC_50_ concentrations. These flasks were kept for 48 h to see the effect of cell death, and untreated cells were considered as the control. All of the cells were collected including the death cells in the tube and other cells detached by trypsinization. These cells were washed with 1X Annexin V binding buffer and centrifuged for 5 min at 600 x g. Then, 5 ul of fluorochrome-conjugated annexin was used to stain cells before incubation for 15 min. All of the cells were again counterstained by PI (1 mg/ml) solution. A cell-containing flow cytometry tube was kept in the dark for a specific time period at a fixed temperature of 4 C before being analyzed by the BD FACSCanto ™ Flow cytometer (Rahman et al., 2021).

#### 2.7.6 Cell cycle assay

FACS is the most popular tool nowadays to identify cell cycle inhibition caused by any inhibitor. A total 1 × 10^5^ number of cells were seeded in different ratio in six-well plates and kept in incubators for attachment. Before treating cancer cells (MCF-7) with the marketed drugs and our selected antagonists, we washed the cells with warmed phosphate buffer saline (PBS). All of the flasks were treated with the different IC_50_ concentrations and kept for 48 h. Then, the cells were collected and centrifuged for 5 min and Next, 700 ul of ice-cold ethanol were used to fix the cells with continuous vortexing. The tubes were kept at −20 C overnight, and the next day, the cells were washed. A total of 500 ul of RNAse and 500ul of propidium iodide were added in each tube and kept in the dark at room temperature. All of the tubes were analyzed using the BD FACSCanto ™ Flow cytometer, and the results were calculated through FACSDiva software (Demir et al., 2018).

#### 2.7.7 Apoptotic gene identification

A qRT-PCR analysis was performed to find the apoptotic gene expression in the extracted RNA sample. Total RNA was isolated using the Purelink RNA mini kits, Thermo Scientific, United States. Before extraction, the cells were treated with the marketed as well as selected drugs and kept for 48 h of incubation. The samples were then mixed with R_1_ and R_2_ and moved to the spin column for centrifugation at 4 C. The RNA was collected and the quality was determined using the NanodropTM spectrophotometer at a wavelength of A260/A280. A revert aid cDNA synthesis kit (Thermo Scientific) was used for cDNA synthesis based on the manufacture’s protocol. The expressions of antiapoptotic genes (BCL-2, BAX, and β-catenin) were determined in the case of treated cells and nontreated cells using the BioRad qRT-PCR machine. GAPDH was used as a reference gene in the case of all cancer cell lines. The gene expression analysis was evaluated by working with TB Green ™ Premix Ex Taq (TAKARA BIO INC.). The Livak (2^−ΔΔCT^) method was used to quantify each gene’s expression.

#### 2.7.8 Enzyme-linked immune sorbent assay test to determine the epidermal growth factor receptor expression

Specific numbers (1 × 10^5^) of cancer cells (A549 and MCF-7) were cultured and treated with our selected compounds (ZINC103239230), gefitinib, or a combination of both ZINC103239230 and gefitinib. The treated cell line was kept in the incubator for 48 h, and the untreated (control) cells were kept for 48 h, 72 h, and 96 h. The supernatant was collected through centrifugation and shifted to the purchased antibody-coated ELISA plate (Solar Bio, China). A total of 100 µl of standards or samples was provided in each well and incubated for 90 min. It was washed four times, and the biotin conjugate antihuman EGFR antibody was added to each well and again incubated for 1 h. A streptavidin working solution was provided to each well, incubated, washed, and 100 µl of substrate was provided for each well. Finally, 50 ul of stop solution was added to each well and the optical density (OD) value was read by the ELISA reader.

#### 2.7.9 Wound healing assay

Both MCF-7 and A549 cells were cultured in six-well plates, and the middle of each well was scratched with a yellow tip in each well. Then, previously prepared antagonists were introduced at IC_50_ concentrations, and the rate of cell migration was then determined.ImageJ software examined the outcomes.

### 2.8 Statistical analysis

The standard error of the mean (SEM) was used to express all data. GraphPad prism, version 7.0 software, was used to perform a one-way analysis of variance to determine how the groups differed from one another. Duncan’s multiple range test (DMRT) was used for the individual comparisons. When *p* < 0.05, values were deemed statistically significant.

## 3 Results

### 3.1 Pharmacophore modeling based on structure

**FIGURE 2:**
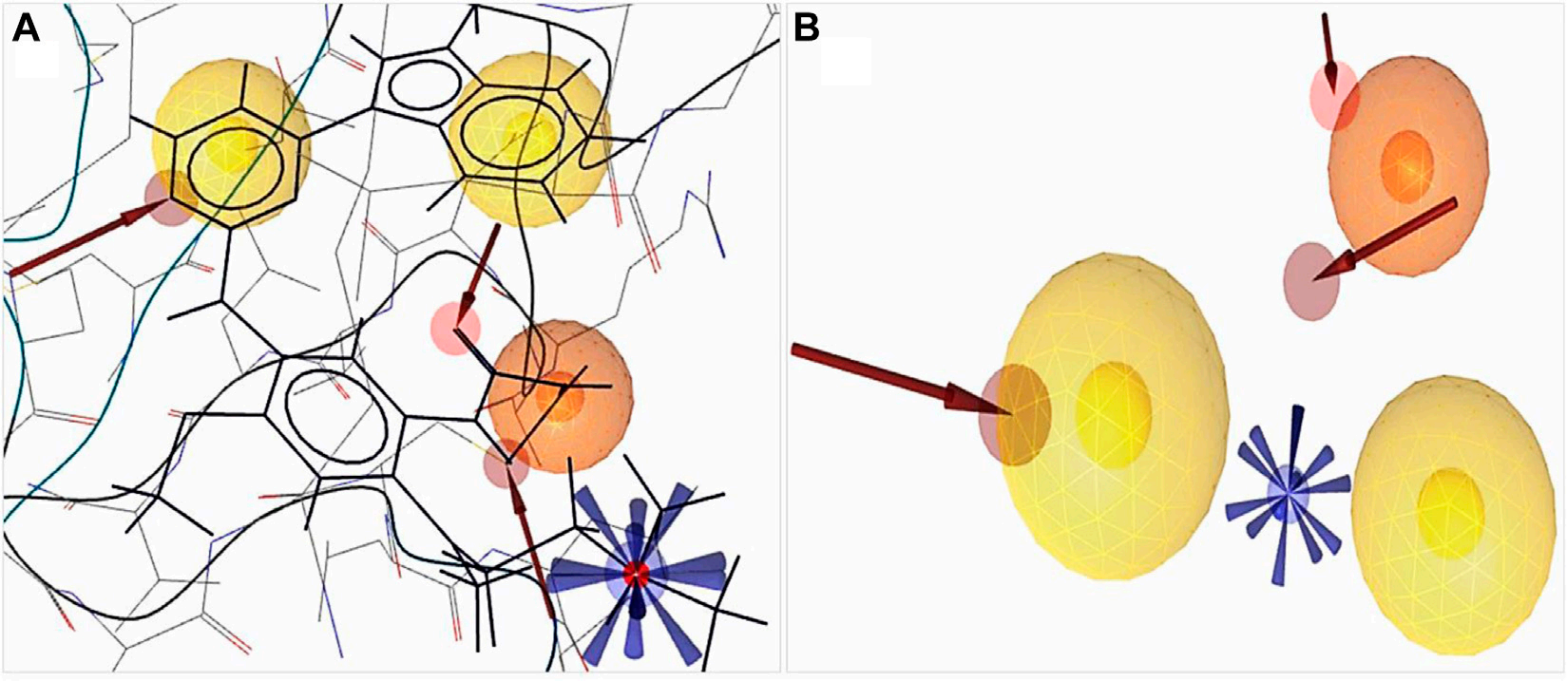
Structure-based pharmacophore model generation. **(A)** 3D structure-based pharmacophore model of protein (PDB ID: 6JXT) in complex with the YY3 ligand was identified from the protein data bank. **(B)** Complex features were merged using ligand scout v4.4 advance tools, three hydrophobic features indicated by a yellow spherical shape 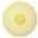, one positive ionizable by a blue star shape 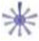, three hydrogen bond acceptors by a red spherical or red arrow shape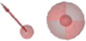 and retrieved excluded volume are not shown in figure.

A 3D pharmacophore model was identified based on several parameters, i.e., source, experimental methods, refinement resolution, release date, and the toxicity result of attached ligands from the protein data bank. EGFR protein (PDB ID: 6JXT) attached with two ligands YY3 and Cl^−^ was selected for docking studies. Protein interactions with ligands were evaluated by the X-ray diffraction method with the resolution, 2.31 Å; R-value free, 0.239, which is significantly less than the standard value of 0.25; R-value work, 0.216; and R-value observed, 0.218. To determine the better inhibitor than YY3, the ligand scout v4.4 advance software was applied to identify main chemical features depending on the pharmacophore model. We detected total 20 features including three hydrophobic (H) interaction features, one positive ionizable (PI) area, three hydrogen bond acceptors (HBAs), and 14 excluded volume features that were not indicated in Figures 2A,B.

The derived crystal structure of our selected protein depicted that the hydrogen bond predominates the interaction of protein and ligand (Supplementary Figure S1). Hydrophobic interactions were formed where benzene rings participated in bond formation with the VAL726, LEU844, ALA743, and ASP800 amino acid residues. A total of three HBAs were formed, the nitrogen of the benzene ring participated with MET793 and HOH1219 residues with the oxygen of the side chain, and the carbon of another benzene ring with ASP800 residues. A positive ionizable pharmacophore feature was also connected to the amino acid ASP800.

### 3.2 Model validation

**FIGURE 3:**
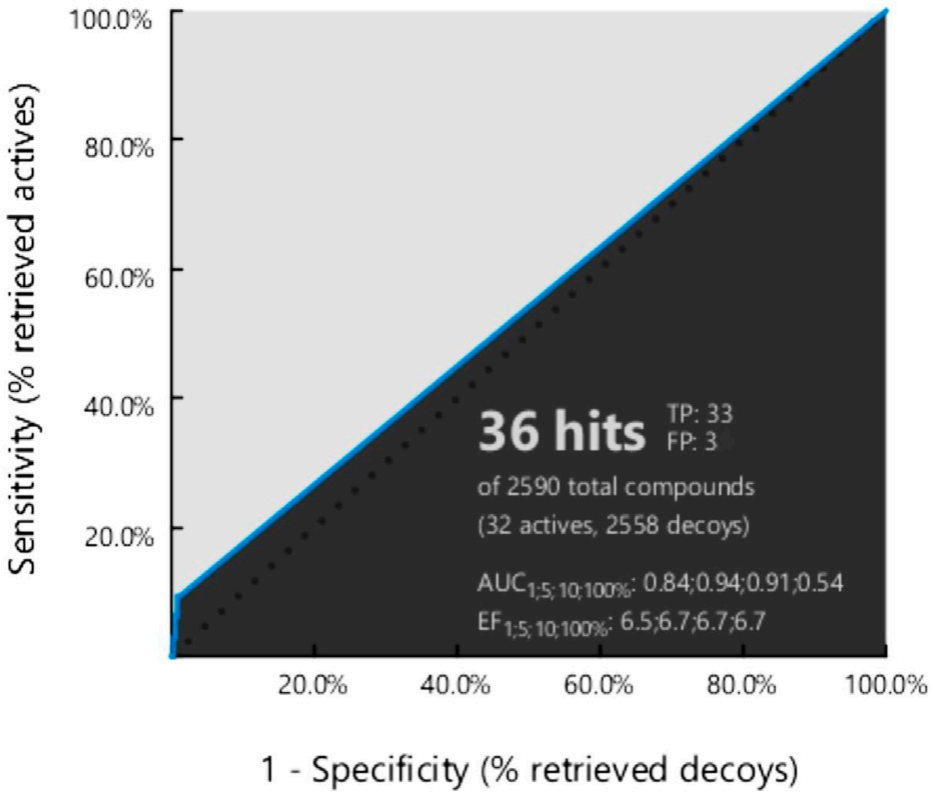
Generated ROC curve based on the active to the target molecule have found 32 actives, and 8,846 decoy compounds were observed.

Our obtained pharmacophore model was subjected to validation using 32 active-known EGFR antagonists (Supplementary Table S2) with the 8,846 decoy molecules obtained through the enhanced DUDE database. After screening completion, the EF value was observed along with the AUC value (Figure 3).

### 3.3 Virtual screening based on the pharmacophore model

#### 3.3.1 Dataset generation

Based on the virtual screening, we can identify active and structurally similar compounds from the ZINC database. This freely accessible database, which has more than 200 million purchasable compounds and substances, can be used for biological assays (Wang et al., 2010; Dain Md Opo et al., 2022). In our study, the structure and other characteristics of the chosen ligands were also determined using this database. This database is enriched with 2D and 3D structures and their boiling points, melting points, molecular weights, crystal structures, toxicity data, and safety precautions. Possible hits were chosen based on the similarity of maximum features. Initially, we searched the ZINC database of natural products, natural derivatives, and purchasable compounds, and a total of 6,000 compounds were retrieved for further screening. Most of the obtained hit compounds were saved for further screening (Irwin et al., 2012).

#### 3.3.2 Virtual screening

Previously obtained compounds were screened using structurally validated pharmacophore features. The generated features from protein ligand interactions were applied to the prepared “idb” format file of natural compounds and shifted to the database. We omitted one feature at the time of starting screening by the ligand scout v4.4 advanced software and retrieved 36 potential hits with a pharmacophore fit score of 65.82 to 55.29, except gefitinib was 74.53. It is not necessary to match all features at the time of screening, and our selected protein had fewer features than others; therefore, the fit score was comparatively low. Usually, the pharmacophore fit score shows a good geometric fit of features to the structure-based pharmacophore model. Based on the obtained pharmacophore fit score, we arranged compounds for further validation (Dain Md Opo et al., 2022).

### 3.4 Molecular docking-based virtual screening

#### 3.4.1 Binding site and grid generation

Binding site identification is an essential part of efficient docking and virtual screening. Proteins typically have multiple pockets that are compatible in size, shape, and suitability for ligand attachment (Yun et al., 2007; Dain Md Opo et al., 2022). The BIOVIA Discovery Studio Visualizer Tool (v16.1.0) was applied to identify the binding site attached with two ligands to the EGFR protein, and the CASTp server was also used to identify the active site of the protein (Supplementary Figure S2). This tool showed that the YY3 ligand of EGFR formed nine van der Waals bonds (ALA859, LEU858, LEU862, TYR869, ALA722, PRO877, VAL876, ARG841, and THR790) and two conventional hydrogen bonds with ASN842 and ASP855. By interacting with the ligand YY3, another amino acid residue, LYS875, formed a carbon hydrogen bond. Moreover, one pi-cation bond with the LYS745 residue, one pi-anion bond with ASP837, one pi-sigma bond with the Val726 residue, one pi–pi T-shaped with PHE723, one alkyl bond with ALA743, and one pi-alkyl bond with LEU844 were formed (Supplementary Figure S2B).

#### 3.4.2 Molecular docking

The most active compounds from the screening of natural compounds were further subjected to docking to get the best binding results. Our epidermal growth factor receptor has a chain with a sequence length of 331 and two unique ligands attached to it. The nucleotide binding positions vary from 718 to 726 and 790 to 791 (Yun et al., 2007; Jura et al., 2009). The filtered molecules were docked by Autodock Vina (version 1.1.2), and before docking, protein as well as ligand molecules were prepared using AutoDockTools. The binding energy obtained after docking depicted the binding activity between our screened molecules and the EGFR protein. The top four compounds were chosen for the next experimental analysis depending on their binding scores, from −9.9 kcal/mol to −9.2 kcal/mol. We obtained the molecular docking score of our chosen compounds (Table 1). When compared to the selected antagonists, compound gefitinib (ZINC19632614) had a lower binding energy (−7.8 kcal/mol).

**TABLE 1:** Four selected compounds including gefitinib based on the docking score and pharmacophore fit score.

Number	Zinc ID	Molecular weight (g/mol)	Docking score (kcal/mol)	Fit score	XlogP3	Molecular formula
1	ZINC96937394	389.8	−9.9	66.42	4.3	C_21_H_16_ClN_5_O
2	ZINC14611940	433.5	−9.6	66.27	3.1	C_23_H_23_N_5_O_4_
3	ZINC103239230	463.6	−9.5	65.82	3.8	C_25_H_33_N_7_O_2_
4	ZINC96933670	455.5	−9.2	66.13	3.6	C_25_H_25_N_7_O_2_
5	ZINC19632614	446.9	−7.8	74.53	4.3	C_22_H_24_ClFN_4_O_3_

#### 3.4.3 Interpretation of protein–ligand binding interaction and pharmacophore modeling

Different pharmacophore fit scores were obtained and a few were excluded depending on the Lipinski rule of five and toxicity analysis. A total of four compounds (ZINC96937394, ZINC14611940, ZINC103239230, and ZINC96933670) showed better binding energies of −9.9 kcal/mol, −9.6 kcal/mol, −9.5 kcal/mol, and −9.2 kcal/mol, respectively, through molecular docking. The van der Waals bonds and hydrogen bonds were the most predominant types of interactions among all of the selected compounds (Supplementary Table S3). Gefitinib, an EGFR tyrosine kinase inhibitor, has also demonstrated favorable protein interactions (Supplementary Figure S3).

**FIGURE 4:**
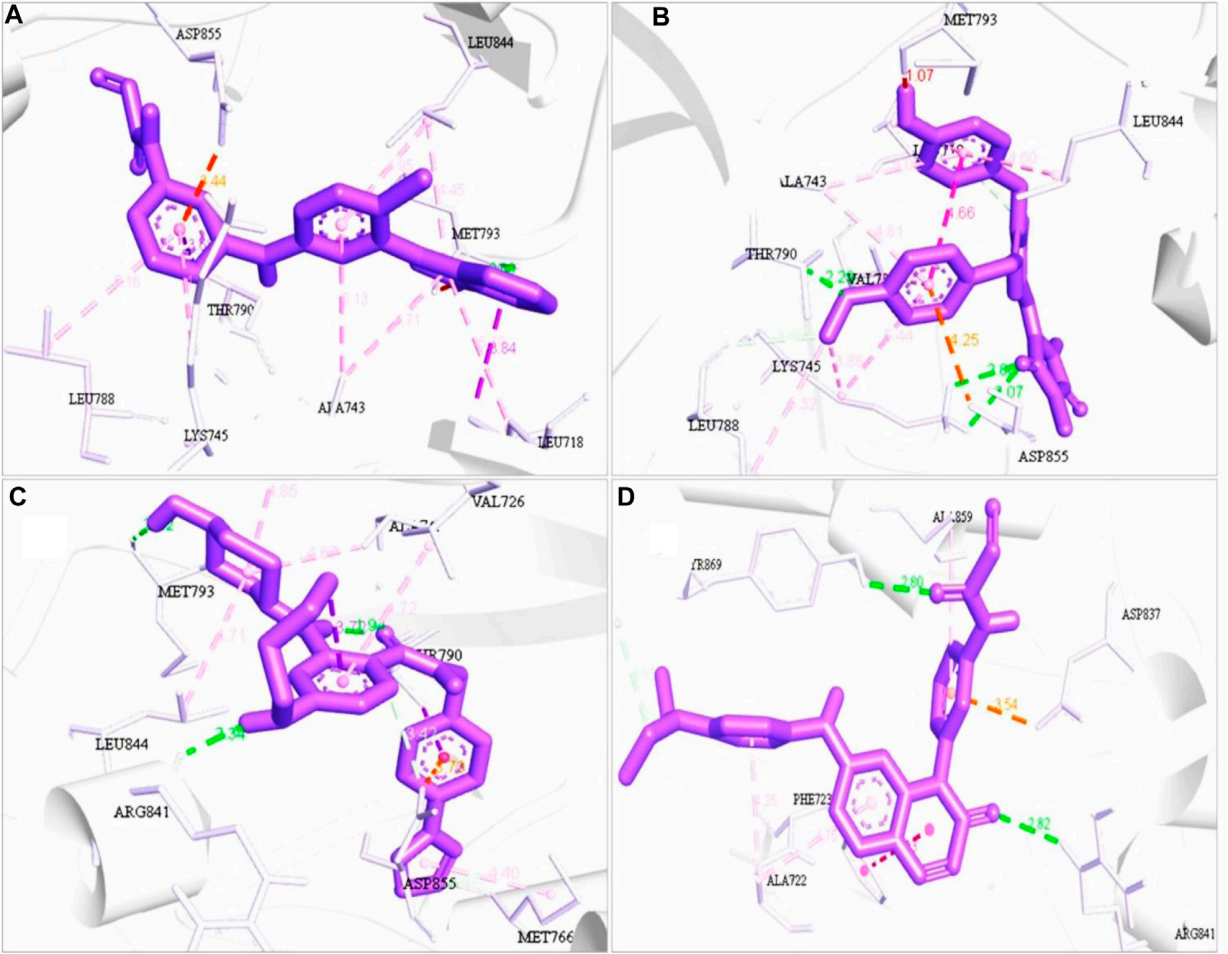
3D structure interaction of **(A)** ZINC96937394, **(B)** ZINC14611940, **(C)** ZINC103239230, and **(D)** ZINC96933670 with the binding site of EGFR protein. The amino acid participation in the interaction are shown in black.

**FIGURE 5:**
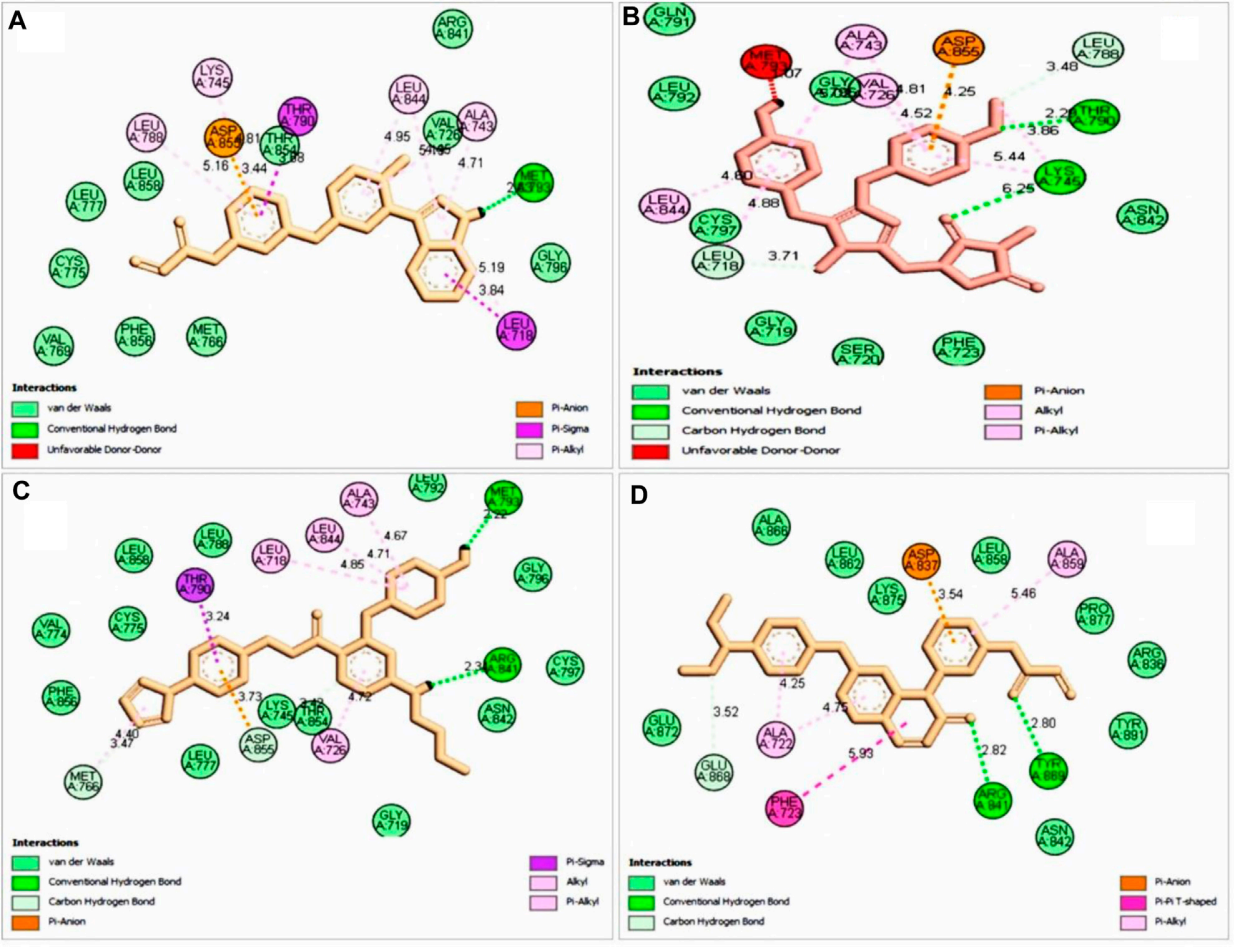
2D interaction with selected ligand **(A)**, ZINC96937394 **(B)**, ZINC14611940 **(C)**, ZINC103239230, and **(D)** ZINC96933670. Bond types are shown in several colors such as blue, red, purple, light pink, deep pink, and green.

The most abundant type of interaction was pi-alkyl interaction, interaction of the pi electron cloud over an aromatic group, and the electron group of an alkyl group. At the same time, van der Waals forces exist for each ligand and protein interaction (Figures 4, 5). All of the natural compounds, ZINC96937394, ZINC14611940, ZINC103239230, and ZINC96933670, were shown to exist as conventional hydrogen bonds and carbon hydrogen bonds with several amino acids, except ZINC96937394, which had a conventional hydrogen bond. In a study of pharmacophore features analysis (Supplementary Figure S4), all compounds were found to have better pharmacophore properties than the selected ligands (Pub Chem ID: 71496458). The 2D features obtained through compound analysis have been mentioned in Supplementary Figure S5.

### 3.5 Pharmacokinetic profile analysis

#### 3.5.1 ADME profile evaluation

Pharmacokinetic parameters are important to determine the drug’s efficacy and toxicity prediction based on drug concentration. The interaction of drug molecules with the target compounds depends on the amount of drug entering in the plasma (Saghir and Ansari 2018) The bioavailability of a drug before going to clinical evaluation can be predicted using software-based analysis (Swiss ADME). The lipophilicity of ZINC103239230 was in the highest position among the other natural compounds that indicated that the compounds could easily diffuse into the body (Table 2).

**TABLE 2:** Prediction of the pharmacokinetic properties of our selected five hit compounds. The drug’s likeliness profile and GI absorption indicated that all can be easily absorbed in the body and have the possibility to produce a quick onset of action.

Property		ZINC96937394	ZINC14611940	ZINC103239230	ZINC96933670
Physico-chemical properties	MW (g/mol)	389.8	433.5	463.6	455.5
	Heavy atoms	28	32	34	34
	Rotatable bonds	5	6	10	8
	H-bond acceptors	4	6	7	7
	H-bond donors	3	2	4	2
	Molar refractivity	112.52	126.74	132.75	134.94
Lipophilicity	Log Po/w	2.89	2.81	3.89	2.66
Water solubility	Class	Poorly soluble	Poorly soluble	Poorly soluble	Poorly soluble
Pharmacokinetics	GI absorption	High	High	High	High
Drug likeliness	Lipinski, violation	Yes	Yes	Yes	Yes
Medi Chemistry	Synthetic accessibility	3.00	3.78	4.29	3.54

#### 3.5.2 Toxicity result prediction

In modern drug discovery, toxicity analysis is an important parameter for getting efficient treatment. Computer-based toxicities are a good choice for the accuracy and cost-effectiveness of the drug and are able to pass the animal model. We used the freely accessible TEST tool v4.2.1 and the ProTox-II server to get these toxicity data (Table 3). The compound ZINC96937394 was able to produce hepatic and immune toxicity and belonged to toxicity class 4. This means that it might not be suitable for oral delivery until there is a modification in dosage form. Again, immunotoxicity and mutagenicity have been identified in the case of natural compounds ZINC96933670 and belong to class 4.

**TABLE 3:** Toxicity screening of our four selected compounds. ZINC14611940 and ZINC103239230 have shown low toxicity in comparison to other compounds.

Endpoint	Target	ZINC96937394	ZINC14611940	ZINC103239230	ZINC96933670
Organ toxicity	Hepatotoxicity	Active	Inactive	Inactive	Inactive
Toxicity endpoints	Carcinogenicity	Active	Inactive	Inactive	Inactive
	Immunotoxicity	Active	Inactive	Active	Active
	Mutagenicity	Inactive	Inactive	Inactive	Active
	Cytotoxicity	Inactive	Inactive	Inactive	Inactive
	LD_50_ (mg/kg)	300	3,200	1,190	660
	Toxicity class	3	5	4	4
Tox21-nuclear receptor signaling pathways	Androgen receptor (AR)	Inactive	Inactive	Inactive	Inactive
	Aryl hydrocarbon receptor (AhR)	Inactive	Inactive	Inactive	Inactive
Tox21-stress response pathway	Heat-shock factor response element	Inactive	Inactive	Inactive	Inactive
Fathead minnow LC50 (96 h)	mg/L	2.67E-02	N/A	0.16	8.76E-03
48-h *Daphnia magna* LC_50_	mg/L	0.24	1.70	53.82	1.31
Developmental toxicity	Value	0.79	0.48	0.41	0.79
Oral rat LD_50_	mg/kg	2349.00	1373.71	1420.37	872.02
Bioaccumulation factor	Log10	0.68	0.65	0.82	N/A

### 3.6 Molecular dynamic simulation

RMSD measurement: the root mean-square deviation of a protein was used to determine structural similarity through the superimposition of two structures and a mathematical movement of a particular atom compared to the standard frame. To evaluate the RMSD of protein, we calculated the Cα backbone, side chain, and heavy atoms of protein based on the selected attached ligand with the reference time (100ns). We used the most popular equation to calculate RMSD for frame x (Eq. 1).

$${RMSD}_{x}=\sqrt{\frac{1}{N}}\overset{N}{\underset{i=1}{\sum }}\left(r_{i}^{\prime }\;\left(t_{x}\right)\right)-\;r_{i\;}{\left(t_{ref})\right)}^{2}.$$ (1)

#### 3.6.1 Protein, ligand root mean-square deviation, root mean-square fluctuations, and radius of gyration analysis

**FIGURE 6:**
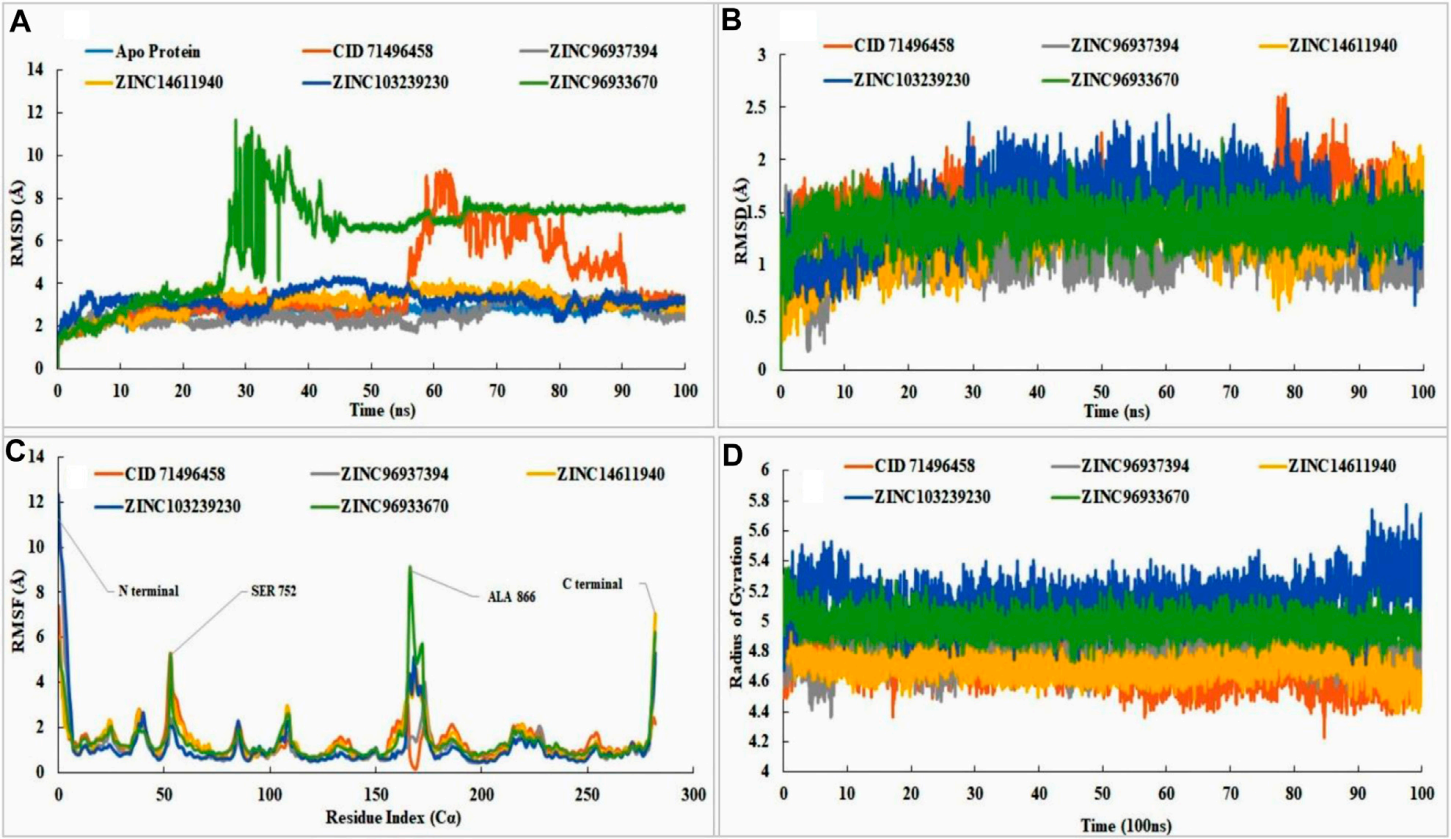
RMSD, RMSF, radius of gyration value of apo-protein, selected ligand (CID71496458) and also the selected four compounds in the complex with EGFR protein. In molecular dynamic simulation, protein RMSD analysis **(A),** the ligand RMSD **(B),** RMSF value analysis **(C),** and radius of gyration **(D)** were analyzed for selected four compounds at 100ns. The simulation was conducted by Schrodinger Maestro software with academic version 2020-3. Different colors indicated several compounds, i.e., apo-protein (blue), CID71496458 (orange), ZINC96937394 (gray), ZINC14611940 (gold), ZINC103239230 (dark blue), and ZINC96933670 (green).

An RMSD value was calculated depending on our selected atom, and this value indicated the structural pattern at the time of simulation. Fluctuations of 1–3 Å were acceptable and an upper value of more than this indicated the instability of the protein. Our protein ligand docking complex simulation results fall within the range of 1–3 Å, with the exception of ZINC96933670, which showed instability from 20 ns to 50 ns but showed stability after 70 ns. The control compound was shown to have instability from 60ns to 90ns and again gradually come down to 3 Å with reliable stability. From the simulation results, three compounds, ZINC14611940, ZINC96937394, and ZINC103239230, showed better stability than the apo-protein. The control ligand (CID: 71496458) showed fluctuations between 60 ns and 90 ns and then came to a stable position (Figure 6A).

The stability of the ligand was rigorously identified through binding with the respect of protein and with the binding pocket. The RMSD of all of the leading compounds, including the control, showed an excellent value with no variations (< 3 Å). Pub Chem ID 71496458 indicated small fluctuations at 80 ns but not more than 3 Å. All of the selected ligands showed stability with respect to the protein and its binding pocket (Figure 6B). Fluctuations revealed that the ligand was capable of diffusing away from the protein binding site. Analysis of RMSF showed the attachment stability of binding with the amino-acid sequence with a specific time period such as the ligand RMSF indicated the interaction of the ligand with the protein residue. Higher peaks showed us the lower amount of stability with the binding residue. Fluctuations are more common at the starting and ending tail positions of N- and C-terminals compared to the other positions. Analysis of the data showed that control compounds showed fewer fluctuations from the start of the N terminal compared to the selected compounds (ZINC103239230 and ZINC96937394), but the fluctuation was high in contrast to the other at 54 ns. ZINC96933670 (green) has shown the highest fluctuations in 166 positions near about although most of the compounds had shown fluctuation at this position except the control. Most of the compounds showed the maximum fluctuations between positions 53-56 (SER, PRO, LYS, and ALA) and 165-173 (ALA, ALA, ALA, GLU, TYR, HIS, ALA, GLU, and LYS). The maximum stability of ZINC103239230 (dark blue) was observed when compared to the other selected compounds in most of the positions. The stability of all compounds was good with slight variations of ZINC96933670 at positions 53 (SER 752) and 166 (ALA 866). Attached ligand (Pub Chem ID: 71496458) showed the least fluctuations in the C-terminal region, and ZINC14611940 (gold) provided the highest fluctuations compared to the other compounds (Figure 6C). The radius of gyration analysis showed that all of the compounds were compressed at 100 nns of simulation time. ZINC103239230 indicated a decrease in compression from 89 nns to 92 nns and again compressed to 100 nns. Usually, the radius of gyration reveals the structural deformation and formation process throughout the simulation time (Figure 6D).

#### 3.6.2 Formation of bond between proteins and ligands

The most common types of bonds in protein–ligand interactions were hydrogen bonds, hydrophobic bonds, ionic bonds, and water bridges. The existence of H-bond influence in pharmacokinetic and pharmacodynamics properties in drug design, ionic bond formation between two oppositely charged ions, which are not usually involved in the H bond (Yunta 2017). In the control, the H bond was predominant in the GLN791 position, but in ZINC14611940, it was the position in ASP837. In ZINC103239230, most existing bonds were hydrophobic in position PHE723. A total of four bonds were present in ZINC96937394 with the amino acid TYR869 position, and ZINC96933670 showed the existence of predominant H bonds and hydrophobic and water bridges at position ARG748 (Supplementary Figure S6).

### 3.7 Analysis of cell death by morphological changes

**FIGURE 7:**
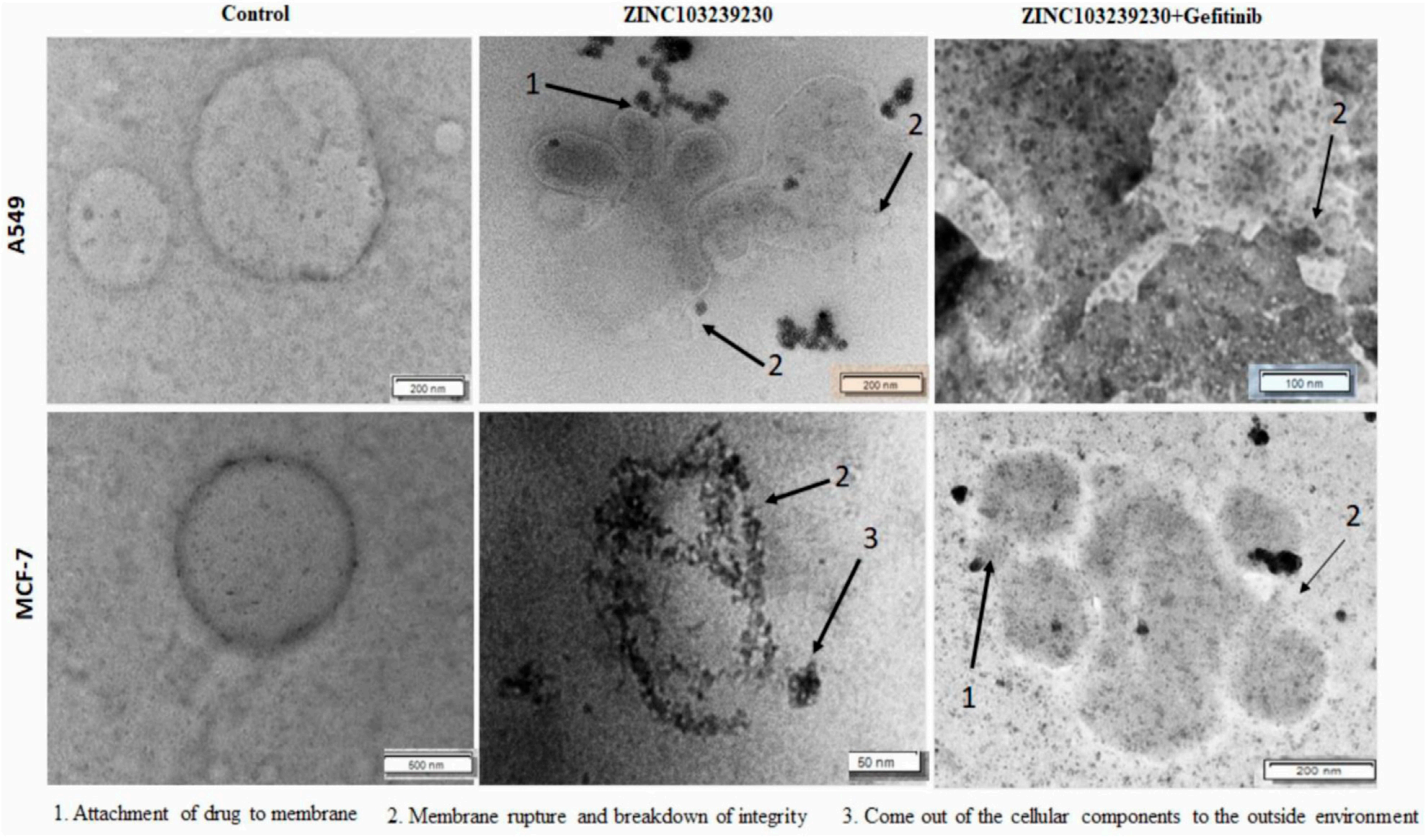
TEM analysis of A549 and MCF-7 cells after 48 h of treatment. Cancer cells were treated by ZINC103239230, gefitinib. (1) Indicates the attachment of drug to the membrane and the penetration, (2) breakdown of the membrane integrity and rupture of the membrane, and (3) breakdown and coming out of nucleus from the cell.

The number of cancer cells was decreased, and they died by changing their shape and through damage to membrane integrity. In lung cancer treatment, ZINC103239230 alone and both gefitinib and ZINC103239230 showed similar structural changes, but gefitinib alone was shown to induce cell death with different structural alterations (Supplementary Figure S7). In MCF-7, gefitinib kills the cell through necrosis. Together, gefitinib and ZINC103239230 were shown to induce cell death. Apoptosis cell death was observed for both A549 and MCF-7 under transmission electron microscopy (Figure 7).

### 3.8 Cell death measurement

**FIGURE 8:**
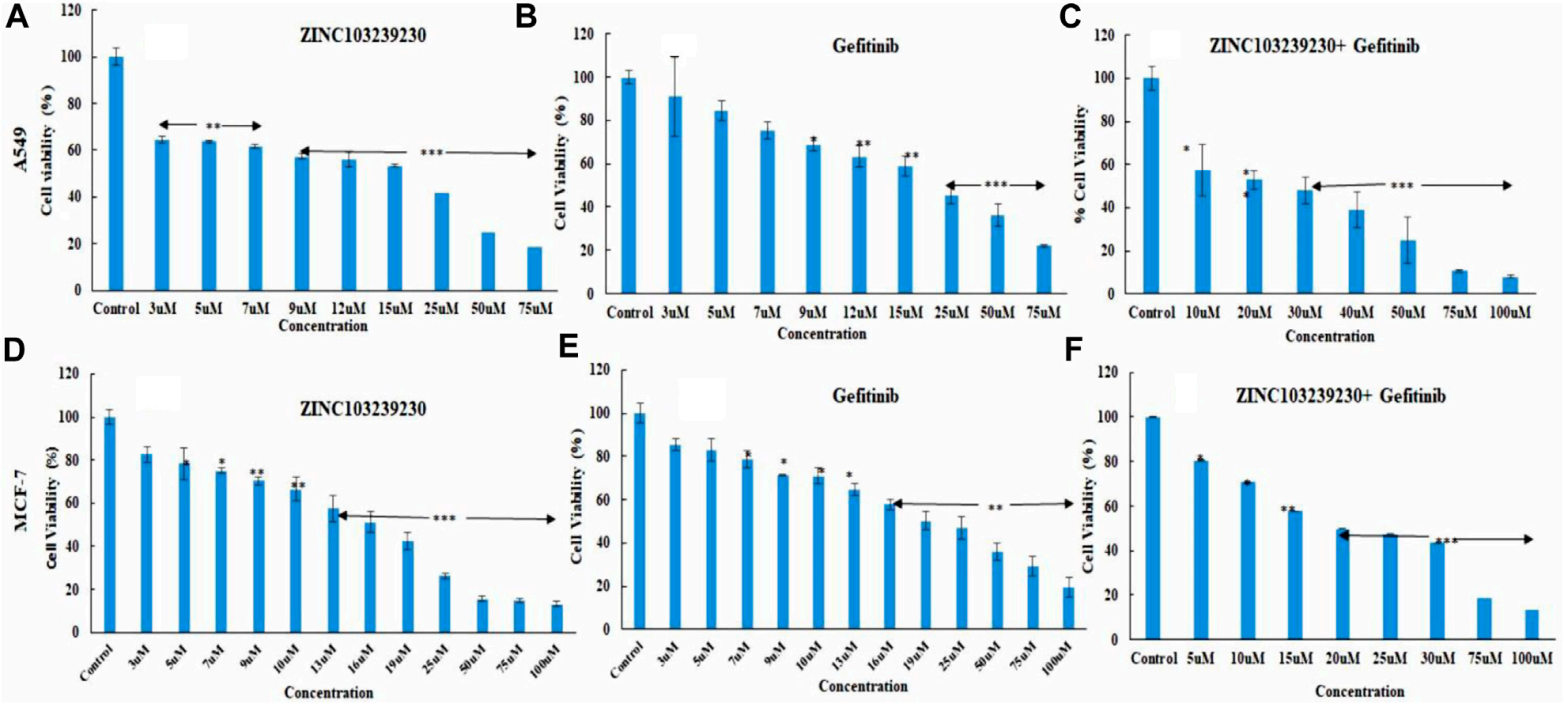
Cell death analysis by MTT assay. In lung cancer cells, ZINC103239230 showed the maximum cell death overall to gefitinib and a combination of both **(A–C)**. In the case of breast cancer (MCF-7), ZINC103239230 showed higher cell death in comparison to the other. The overall cell death (A549, MCF-7) ZINC103239230 > combination > gefitinib.

The cytotoxicity of our selected drugs (ZINC103239230, gefitinib, and their combinations) was determined using several concentrations to measure 50% cell death of each product. The IC_50_ for gefitinib (15.6206 µM), ZINC103239230 (14.8102 µM), and for the combination of gefitinib and ZINC103239230 (13.3082 µM) in MCF-7 treatment was determined (Figures 8D–F). The maximum inhibition (ZINC103239230: 86.91%) of cell death was observed in comparison to gefitinib and its combination. The inhibition of our selected compound, ZINC103239230, was shown to have more effects compared to the marketed drug. In the case of MCF-7 inhibition, the combination of gefitinib and ZINC103239230 also demonstrated a synergistic effect. In the inhibition of A549 lung cells, ZINC103239230 compound showed 50% cell death (IC_50_) at 10 µM and gefitinib showed cell death at 13.062 µM. The combination of ZINC103239230 and gefitinib was shown to be 12.738 µM (Figures 8A–C). Graphpad Prism v9.0 was used to calculate the IC_50_ value.

### 3.9 Gene expression analysis

**FIGURE 9:**
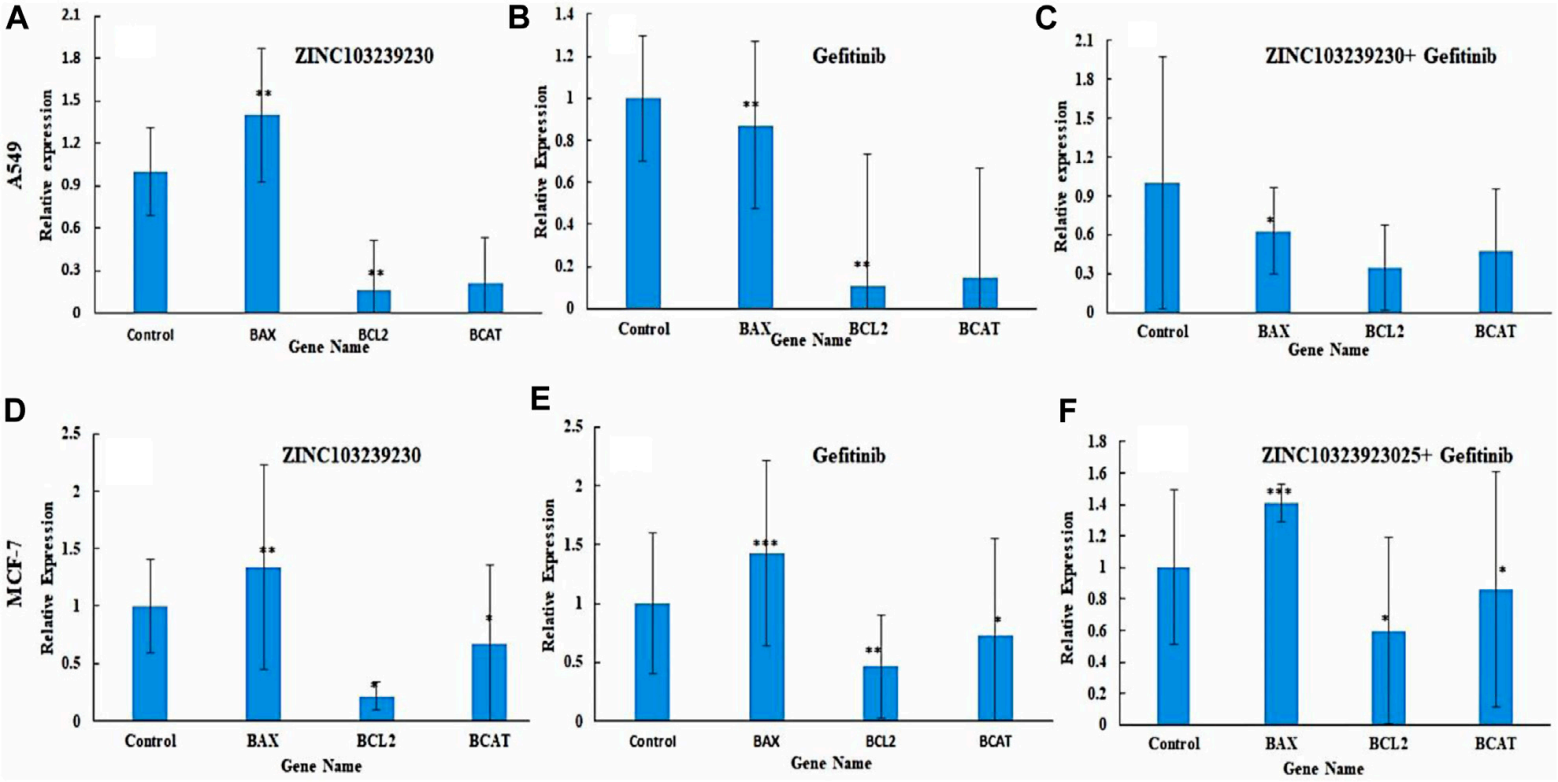
Expression of apoptotic genes in two different cell lines: A549 **(A–C)** and MCF-7 **(C–E)**. Bax expression was slightly lower in gefitinib **(B)** and combination **(C)** compared to ZINC103239230 **(A)**. In MCF-7, apoptotic genes showed better expression of ZINC103239230 **(D)** in contrast to others **(E,F)**.

The expression of several antiapoptotic genes was identified by qRT-PCR analysis. Treatment of A549 and MCF-7 by our selected antagonist (ZINC103239230) with IC_50_ concentration showed the best results compared to gefitinib and its combination. The expression of these genes demonstrated the induction of apoptosis with the presence of all our compounds. The expression of all genes (BAX, BCL-2, and β-catenin) indicated the apoptotic cell death of MCF-7 cells and the intracellular BCL-2 downregulation, selected compound showed the best result in comparison to the others (gefitinib, gefitinib + ZINC103239230). In the case of BAX expression, gefitinib and gefitinib + ZINC103239230 indicated the same expression compared to ZINC103239230 that showed less expression. In a comparison of gefitinib and ZINC103239230 treated MCF-7 cell lines, both gefitinib and ZINC103239230 combination treatments showed a slightly upregulated catenin. The induction of apoptosis by the treatment of gefitinib, ZINC103239230, and both gefitinib and ZINC103239230 were defined by BAX, BCL-2, and β-catenin gene against the A549 cell line. The BAX gene was upregulated in ZINC103239230, compared to gefitinib and both. The Bcl-2 expression was downregulated for all treatments, but slightly less for both gefitinib and ZINC103239230. Our selected compound showed that the overall apoptotic gene expression was high in comparison to the marketed product gefitinib and the combination of both (Figure 9).

### 3.10 Epidermal growth factor receptor expression measurement

**FIGURE 10:**
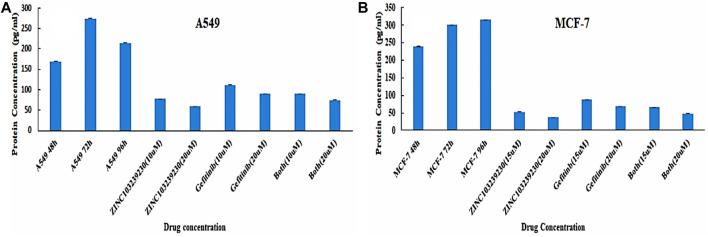
Quantitative sandwich-based ELISA for lung cancer (A549) and breast cancer (MCF-7) cell lines. **(A)** EGFR concentration determination in A549 and **(B)** EGFR measurement in MCF-7 supernatant.

An enzyme-linked immunosorbent assay was used to detect the EGFR protein expression with our treated drugs. The results indicated a decrease in EGFR expression compared to the control (Figure 10). The control protein concentration (A549) was increased with the increase of time: 48 h (169.3043 pg/ml), 72 h (274.7391 pg/ml), and 96 h (214.5217 pg/ml). ZINC103239230 was shown to reduce 78.13043 pg/ml and 59.6087 pg/ml at two different concentrations (10µM and 20 µM). The combination (ZINC103239230 + gefitinib) decreased EGFR concentration to 74.6087 pg/ml (20 µM), and gefitinib was shown to decrease the concentration to 89.695 pg/ml (20 µM). In MCF-7, ZINC103239230 showed a maximum reduction of 37.7826 pg/ml (20 µM). The combination of both expressed more efficient reduction of EGFR at 47.8913 pg/ml (20 µM), whereas the gefitinib 20 µM reduced 68.434 pg/ml. The EGFR control graph has been provided in Supplementary Figure S8.

### 3.11 Cell death measurement by flow cytometry

**FIGURE 11:**
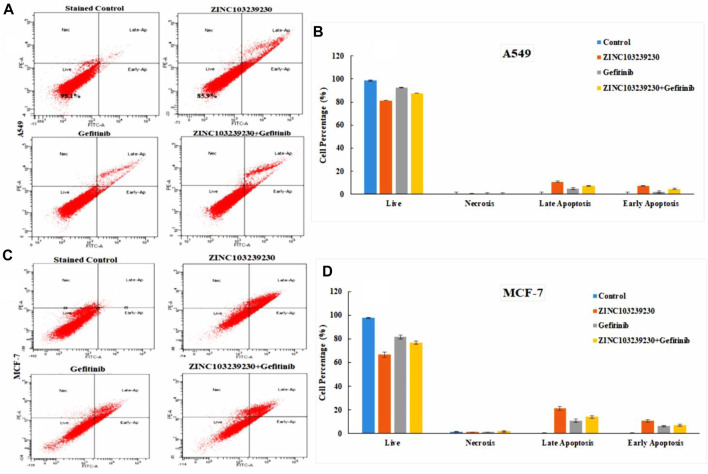
Apoptosis cell death was measured by flow cytometry. BD FACSDiva software was used to calculate the cell death for both A549 **(A)** and MCF-7 **(C)** cell lines. **(B,D)** Cell death analysis data that accurately quantify the fraction of cells in each phase.

This method involves detecting phosphatidylserines in apoptotic cells and assessing them as annexin V-FITC binds to phosphatidylserine (Lee et al., 2013). The total cell death in A549 (Figures 11A,B) was measured at 1.9% in the case of the untreated control. In ZINC103239230, the early apoptosis was increased (5.4%) using the IC_50_ concentration after 48 h, and meanwhile, gefitinib showed a higher late apoptosis (3.8%), whereas the early apoptosis was 1.7% after 48 h treatment with the same concentration. In contrast, the percentage of cells entering into early apoptosis was reduced to 3.6% in comparison to ZINC103239230 alone used using the IC_50_ concentration. However, the early apoptotic cell death was higher in the case of our selected antagonist (ZINC103239230). In the MCF-7 cell line, compound ZINC103239230 showed the that early apoptosis was 10.5%, whereas the marketed drug (gefitinib) showed the half of early apoptosis (5.8%) after 48 h of incubation (Figures 11C,D). For combination treatment, early apoptosis was 6.4% and late apoptosis was 12.7%. Therefore, the apoptosis for ZINC103239230 was sharply increased (31.9%) in comparison to both gefitinib (17.1%) and combination treatment (21.3%).

### 3.12 Analysis of cell cycle

**FIGURE 12:**
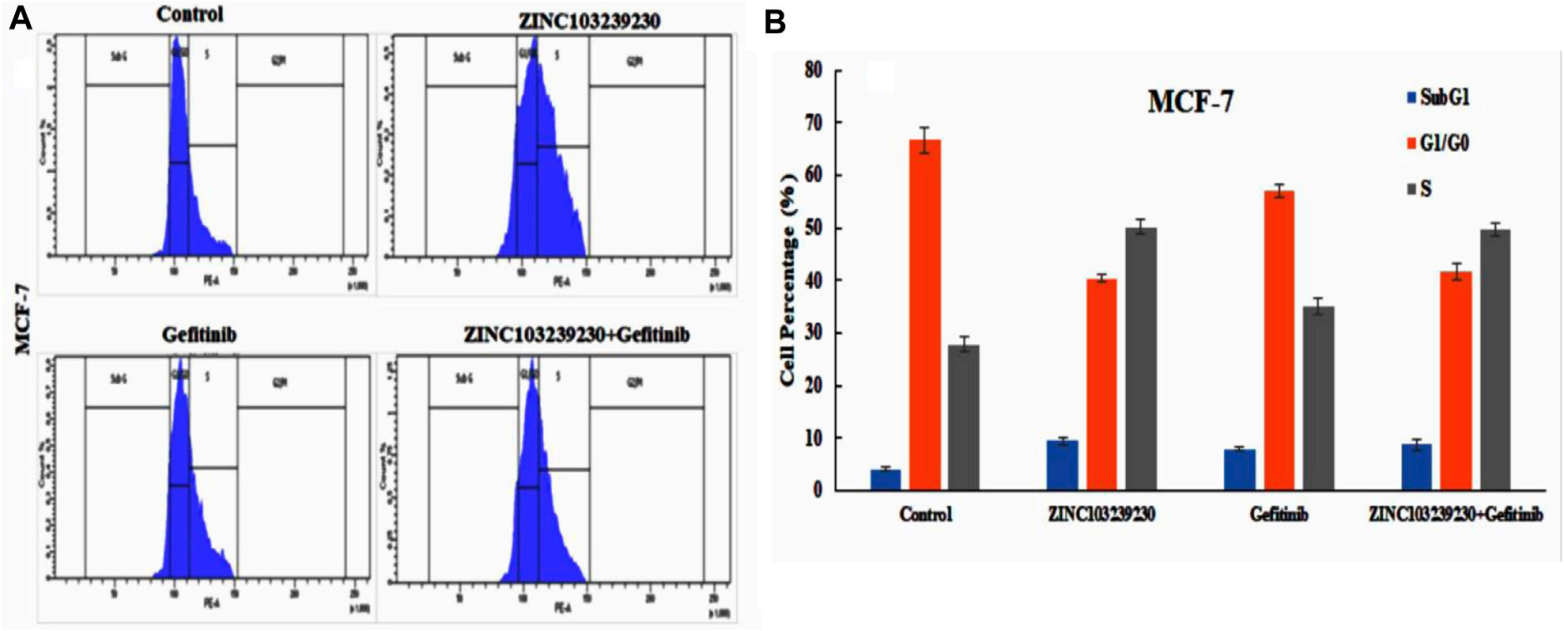
Effects of ZINC103239230, gefitinib and combination blocked the S phase of the cell cycle in the MCF-7 cell line.

The cell cycle analysis revealed that our compound was more active against the MCF-7 cell line and had a lower effect on the cell cycle death of the A549 cell line. The SubG1 phase (9.46%) was higher in ZINC103239230, and the accumulation of cells increased in the S phase in comparison to the G0/G1 phase. The S phase for combination treatment was also more than the gefitinib used alone (Figures 12A,B).

### 3.13 Cell migration analysis

**FIGURE 13:**
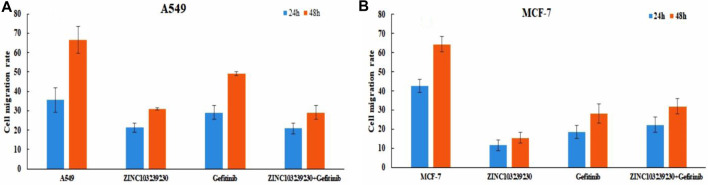
Cell migration analysis for both lung cancer and breast cancer cell line. ImageJ software was used to calculate the migration area.

In our study, the untreated (control) group (MCF-7, A549) migrated the scratched blank area more rapidly in comparison to the treated group (Supplementary Figure S9). In MCF-7 (Figure 13B), the selected compound ZINC103239230 has shown the lowest migration (ZINC103239230 > Gefitinib > ZINC103239230 + Gefitinib). At the same time, ZINC103239230 also showed a lower migration rate to the scratch area but not more than the MCF-7 cell line in case of the A549 cancer cell treatment (Figure 13A). The overall migration rate was decreased in the lung cancer cell line through the incubation of 24h and 48h based on our compounds' treatment alone or in combination.

## 4 Discussion

EGFR plays an important role in the progression and development of several cancers in humans, on an average 50–60% of lung adenocarcinoma, colon cancer, and breast carcinoma (Pabla et al., 2015; Sigismund et al., 2018). Many medications are permitted alone or in combination with chemotherapy for colorectal cancer, non-small-cell lung cancer, and breast cancer due to the success of EGFR-targeting in the treatment of cancer (Wykosky et al., 2011; Chan et al., 2017; Thomas and Weihua 2019). The majority of the mutations belonged to nonsmokers and the incidence rate is higher among the females than males in the occurrence of lung carcinoma (Fukuoka et al., 2011). In lung cancer eradication, gefitinib binds with the EGFR tyrosine kinase pocket through competitive inhibition to produce the inhibitory activity (Morin 2000). Gefitinib is the good choice for the initial start of the EGFR mutated lung cancer treatment, although 50% of lung cell carcinomas showed resistance to gefitinib at the time of 7–12 months of treatment through mutations L858R and T790M and indicated the upregulation of EGFR activity (Balak et al., 2006; Yun et al., 2008; Chan et al., 2020; Han et al., 2021). Erlotinib, gefitinib, afatinib, and lapatinib are examples of first-generation drugs, also known as kinase inhibitors, that have been shown to be effective against several carcinomas. Due to their effective clinical results against breast, colon, and lung cancer, first-generation EGFR kinase inhibitors continue to be the most widely used medications (Yan et al., 2020). These first-generation TK inhibitors (TKIs) were found to be effective in the treatment of advanced non-small-cell lung cancer (NSCLC) in somatic activating mutations (Martinez-Marti et al., 2019). Gefitinib works against breast cancer through attaching to the ATP-binding site of the EGFR protein (Masuda et al., 2012). The use of gefitinib along with tamoxifen was able to treat metastatic breast cancer and was effective in killing tamoxifen-resistant tumor cells (Zhang et al., 2015). Aspirin combined with gefitinib or osimertinib was shown to able to induce apoptosis of lung cancer cells, and the breast cancer cell percentage was also decreased when aspirin and tamoxifen were combined *in-vitro* and *in-vivo* (Li et al., 2020).

The second-generation of EGFR tyrosine kinase inhibitors, such as afatinib and dacomitinib, are effective against EGFR-mutated breast and lung cancer, but ineffective against T790M-mutated cancer (Abourehab et al., 2021). As a result, third-generation EGFR kinase inhibitors such as WZ4002, CO-1686, and AZD9291 are in clinical trials to target the T790M mutation. The study showed that the EGFRm + sensitizing and T790M resistance mutations are both effectively and selectively inhibited by new oral compound AZD9291 (Yan et al., 2020). The use of TK inhibitors resulted in significant toxicities such as gastro-intestinal disorder, skin rash, diarrhea, and other complications (Hirsh 2011). Bommu et al. (2019) identified potential lead compounds through a computer-aided drug approach against EGFR protein based on the QASR modeling. Several studies were also carried out to identify potential lead EGFR antagonists using computer-aided drug design, and one *in-vitro* study was conducted with different databases screening without any comparative analysis with the currently available drugs in use for treatment, as well as without evaluating the toxicity profile, protein–ligand stability analysis *via* dynamic simulation (Sangande et al., 2020; Weng et al., 2022). For the identification of lead compounds against a specific target, computer-based drug design has emerged as a powerful technique due to the ability to determine the stereochemical quality, toxicity, ligand selectivity, and screening of compound libraries (Song et al., 2009; Baig et al., 2016). In the present study, we investigated the discovery of potential lead compounds using computational drug discovery based on molecular dynamic simulation, toxicity analysis, and determining the binding affinity. The toxicity profile was assessed using the Protox II, Swiss ADME, and the Test Tool. Initially, a pharmacophore feature was generated using the ligand scout v4.4 advanced software. The selected ligand features have been shifted to the ZINCPharmer database and the provided library was screened based on the molecular weight (500 kDa), RMSD less than or equal to 1, to get the structurally similar compounds. The ZINCPharmer database is reached with the natural product library, natural derivative library, and purchasable compound library with more than 18 million compounds (Koes and Camacho 2012). All of the obtained compounds have been shifted to the ligand scout v4.4 advanced software, and a total of 36 antagonists were identified with a maximum fit score of 65.82. The compounds were selected based on the docking score and the toxicity class. Current third-generation inhibitors, osimertinib, belong to class 3 (LD_50_:100 mg/kg) with active immunotoxicity, and first-generation gefitinib belongs to class 5 (LD_50_:2935 mg/kg); both are being widely used as TK inhibitors in Asia (Rahman et al., 2022). These drugs also have hepatotoxicity and immunotoxicity and are active in binding aryl hydrocarbon receptors. Our selected compound belongs to class 4 (LD_50_:1190 mg/kg) and was only active in immunotoxicity.

The morphological study of MCF-7 and A549 revealed that the cancer cell died through breakdown of the membrane with our selected compound and also with the currently used drugs, but both in different ways. The maximum number of cell deaths was observed with the increase of concentration (75 µM and 100 µM) and the IC_50_ showed that the maximum number of cell deaths (30.8%) was observed in the case of MCF-7 by flow cytometry. The EGFR protein concentration was also reduced in breast cancer (MCF-7) more than in the lung adenocarcinoma cell line (A549). Cell migration to the scratch area has been reduced using ZINC103239230 and also a combination of gefitinib and ZINC103239230. Previous studies have been conducted to overcome the resistance of the EGFR targeted drugs, but our study showed the potential to find new lead compounds to overcome the EGFR-TKI mutation (T790M) (Bommu et al., 2017). The pharmacophore model-based drug discovery together with the molecular lab-based studies might be useful to facilitate extensive research for other scientists on EGFR mutation through targeting other EGFR-related carcinomas and can be able to find new candidates with a lower toxicity profile. Current potential lead compounds identification based on the *in silico* and *in-vitro* studies for EGFR overexpressed carcinoma that may have more robust pharmacological effects than currently available marketed drugs. Our novel compounds could target EGFR-related cancers and can reduce the severity of several cancers, including lung and breast cancer. Further *in-vitro* studies and clinical trials may be necessary to develop this potential lead compound against carcinoma.

## 5 Conclusion

Structure-based drug design is becoming increasingly important, effective, and necessary to identify inhibitory drugs against a certain biological target. Based on in-silico drug design, scientists are attempting to find more effective compounds against the EGFR protein with the goal of overcoming current mutations in the case of observed carcinoma treatment. In our study, we identified a potential lead compound against EGFR protein using pharmacophore model-based drug design and molecular biology experimental analysis. The cytotoxicity and flow cytometry studies revealed that our selected antagonist (ZINC103239230) had more antitumor effects in *in-vitro* studies at several concentrations and with an observed IC_50_ value. The EGFR protein concentration in the serum of a cultured cell line revealed the decrease of target protein concentration in comparison to the marketed drug gefitinib. Through an electron microscopy study, the structural changes of both MCF-7 and A549, as well as their death pattern, were expressed using a specific concentration. According to ADME analysis, our compound has better orally bioavailable drug-like properties and lower toxicities than third-generation TK inhibitors. All these findings show that in the future, this selected compound may have better anticancer properties for EGFR-targeted treatment in breast cancer and lung cancer.

## Data Availability

The original contributions presented in the study are included in the article/Supplementary Material; further inquiries can be directed to the corresponding authors.

## Abbreviations

ADMET, absorption, distribution, metabolism, excretion, and toxicity; ATP, adenosine triphosphate; AUC, area under curve; CAAD, computer-aided drug discovery; CASTp, computed atlas of surface topography of protein; CRC, colorectal cancer; DUDE, directory of useful decoys enhanced; EGFR, Epidermal Growth Factor Receptor; ELISA, enzyme linked immune sorbent assay; FDA, food and drug administration; FITC, fluorescein isothiocyanate; IC_50_, inhibitory concentration; IDB, Intermediate Debug File; KRAS, Kirsten rat sarcoma viral oncogene homolog; LC_50_, lethal Concentration 50; LD_50_, lethal Dose 50; MM-GBSA, molecular mechanics with generalized born and surface area solvation; MAPK, mitogen-activated protein kinase; MET, mesenchymal–epithelial transition; PBS, phosphate buffer saline; qRT-PCR, quantitative real time polymerase chain reaction; ROC, receiver operative curve; RMSD, root mean square deviation; RMSF, root mean square fluctuations; SDF, structure data file; SSE, simulation support environment.

## References

[B1] Abourehab M. A. S. Alqahtani A. M. Youssif B. G. M. Gouda A. M. (2021). Globally approved EGFR inhibitors: Insights into their syntheses, target kinases, biological activities, receptor interactions, and metabolism. Molecules 26 (21), 6677. 10.3390/MOLECULES26216677 34771085 PMC8587155

[B2] Aljahdali M. O. Rahman Molla M. H. Ahammad F. (2021). Compounds identified from marine mangrove plant (Avicennia alba) as potential antiviral drug candidates against WDSV, an in-silico approach. Mar. Drugs 19 (5), 253. 10.3390/MD19050253 33925208 PMC8145693

[B3] Baig M. H. Ahmad K. Roy S. Ashraf J. M. Adil M. Siddiqui M. H. (2016). Computer aided drug design: Success and limitations. Curr. Pharm. Des. 22 (5), 572–581. 10.2174/1381612822666151125000550 26601966

[B4] Balak M. N. Gong Y. Riely G. J. Somwar R. Li R. A. Zakowski M. F. (2006). Novel D761Y and common secondary T790M mutations in epidermal growth factor receptor-mutant lung adenocarcinomas with acquired resistance to kinase inhibitors. Clin. Cancer Res. 12 (21), 6494–6501. 10.1158/1078-0432.CCR-06-1570 17085664

[B5] Banerjee P. Eckert A. O. Schrey A. K. Preissner R. (2018). ProTox-II: A webserver for the prediction of toxicity of chemicals. Nucleic Acids Res. 46 (W1), W257–W263. 10.1093/nar/gky318 29718510 PMC6031011

[B6] Bartholomeusz C. Yamasaki F. Saso H. Kurisu K. Hortobagyi G. N. Ueno N. T. (2011). Gemcitabine overcomes erlotinib resistance in EGFR overexpressing cancer cells through downregulation of akt. J. Cancer 2 (1), 435–442. 10.7150/JCA.2.435 21850211 PMC3157020

[B7] Beaumont C. Young G. C. Cavalier T. Young M. A. (2014). Human absorption, distribution, metabolism and excretion properties of drug molecules: A plethora of approaches. Br. J. Clin. Pharmacol. 78 (6), 1185–1200. 10.1111/BCP.12468 25041729 PMC4256609

[B8] Bemanian V. Sauer T. Touma J. Lindstedt B. A. Chen Y. Ødegård H. P. (2015). The epidermal growth factor receptor (EGFR/HER-1) gatekeeper mutation T790M is present in European patients with early breast cancer. PLOS ONE 10 (8), e0134398. 10.1371/JOURNAL.PONE.0134398 26267891 PMC4534377

[B9] Berman H. M. Westbrook J. Feng Z. Gilliland G. Bhat T. N. Weissig H. (2000). The protein data bank. Nucleic Acids Res. 28 (1), 235–242. 10.1093/NAR/28.1.235 10592235 PMC102472

[B10] Bethune G. Drew B. Ridgway N. Xu Z. (2010). Epidermal growth factor receptor (EGFR) in lung cancer: An overview and update. J. Thorac. Dis. 2 (1), 48–51. 10.3978/j.issn.2072-1439.2010.02.01.017 22263017 PMC3256436

[B11] Bivona T. G. Hieronymus H. Parker J. Chang K. Taron M. Rosell R. (2011). FAS and NF-?b signalling modulate dependence of lung cancers on mutant EGFR. Nature 471 (7339), 523–526. 10.1038/NATURE09870 21430781 PMC3541675

[B12] Bommu U. D. Konidala K. K. Pabbaraju N. Yeguvapalli S. (2017). Ligand-based virtual screening, molecular docking, QSAR and pharmacophore analysis of quercetin-associated potential novel analogs against epidermal growth factor receptor. J. Recept. Signal Transduct. Res. 37 (6), 600–610. 10.1080/10799893.2017.1377237 28958213

[B13] Bommu U. D. Konidala K. K. Pabbaraju N. Yeguvapalli S. (2019). QSAR modeling, pharmacophore-based virtual screening, and ensemble docking insights into predicting potential epigallocatechin gallate (EGCG) analogs against epidermal growth factor receptor. J. Recept. Signal Transduct. Res. 39 (1), 18–27. 10.1080/10799893.2018.1564151 31223050

[B14] Bommu U. D. Konidala K. K. Pamanji R. Yeguvapalli S. (2018). Computational screening, ensemble docking and pharmacophore analysis of potential gefitinib analogues against epidermal growth factor receptor. J. Recept. Signal Transduct. Res. 38 (1), 48–60. 10.1080/10799893.2018.1426603 29369008

[B15] Bowers K. J. Chow E. Xu H. RonDrorEastwood O. M. P. Gregersen B. A. Klepeis J. L. (2006). “Scalable algorithms for molecular dynamics simulations on commodity clusters,” in SC ’06: Proceedings of the 2006 ACM/IEEE Conference on Supercomputing, Tampa, FL, USA, 11-17 November 2006.

[B16] Brylinski M. (2017). “Local alignment of ligand binding sites in proteins for polypharmacology and drug repositioning,” in Methods in molecular biology (Totowa, New Jersey, United States: Humana Press), 1611, 109–122. 28451975 10.1007/978-1-4939-7015-5_9 PMC5513668

[B17] Chan D. L. H. Segelov E. RachelWong S. H. Smith A. Herbertson R. A. Li B. T. (2017). Epidermal growth factor receptor (EGFR) inhibitors for metastatic colorectal cancer. Cochrane Database Syst. Rev. 2017 (6), CD007047. 10.1002/14651858.CD007047.PUB2 PMC6481896 28654140

[B18] Chan O. S. H. Lam K. C. Li J. Y. C. Choi F. P. T. Wong C. Y. H. Chang A. T. Y. (2020). Atom: A phase II study to assess efficacy of preemptive local ablative therapy to residual oligometastases of NSCLC after EGFR TKI. Lung Cancer 142, 41–46. Amsterdam, Netherlands. 10.1016/J.LUNGCAN.2020.02.002 32088604

[B19] Dain Md Opo F. A. Alsaiari A. A. Molla M. H. R. Afsar Ahmed Sumon Md Yaghmour K. A. Ahammad F. (2022). Identification of novel natural drug candidates against BRAF mutated carcinoma; an integrative *in-silico* structure-based pharmacophore modeling and virtual screening process. Front. Chem. 0, 1209. 10.3389/FCHEM.2022.986376 PMC9577413 36267655

[B20] Dallakyan S. Olson A. J. (2015). Small-molecule library screening by docking with PyRx. Methods Mol. Biol. 1263, 243–250. 10.1007/978-1-4939-2269-7_19 25618350

[B21] Davies M. Nowotka M. George P. Dedman N. Gaulton A. Atkinson F. (2015). ChEMBL web services: Streamlining access to drug discovery data and utilities. Nucleic Acids Res. 43 (W1), W612–W620. 10.1093/NAR/GKV352 25883136 PMC4489243

[B22] Demir S. Turan I. Aliyazicioglu R. Yaman S. O. Aliyazicioglu Y. (2018). Primula vulgaris extract induces cell cycle arrest and apoptosis in human cervix cancer cells. J. Pharm. Anal. 8 (5), 307–311. 10.1016/J.JPHA.2018.05.003 30345144 PMC6190528

[B23] Engelman J. A. Zejnullahu K. Mitsudomi T. Song Y. Hyland C. Park O. (2007). MET amplification leads to gefitinib resistance in lung cancer by activating ERBB3 signaling. Sci. (New York, N.Y.) 316 (5827), 1039–1043. 10.1126/SCIENCE.1141478 17463250

[B24] Fukuoka M. Wu Y. L. Thongprasert S. Sunpaweravong P. Leong S. S. Sriuranpong V. (2011). Biomarker analyses and final overall survival results from a phase III, randomized, open-label, first-line study of gefitinib versus carboplatin/paclitaxel in clinically selected patients with advanced non-small-cell lung cancer in Asia (IPASS). J. Clin. Oncol. 29 (21), 2866–2874. 10.1200/JCO.2010.33.4235 21670455

[B25] Han R. Jia Y. Li X. Zhao C. Zhao S. Liu S. (2021). Concurrent use of metformin enhances the efficacy of EGFR-TKIs in patients with advanced EGFR-mutant non-small cell lung cancer-an option for overcoming EGFR-TKI resistance. Transl. Lung Cancer Res. 10 (3), 1277–1291. 10.21037/TLCR-20-1153 33889509 PMC8044488

[B26] Hirsh V. (2011). Managing treatment-related adverse events associated with egfr tyrosine kinase inhibitors in advanced non-small-cell lung cancer. Curr. Oncol. 18 (3), 126–138. 10.3747/CO.V18I3.877 21655159 PMC3108866

[B27] Huang S. Hölzel M. Knijnenburg T. Schlicker A. Paul R. McDermott U. (2012). MED12 controls the response to multiple cancer drugs through regulation of TGF-β receptor signaling. Cell. 151 (5), 937–950. 10.1016/J.CELL.2012.10.035 23178117 PMC3672971

[B28] Hughes J. P. Rees S. S. Kalindjian S. B. Philpott K. L. (2011). Principles of early drug discovery. Br. J. Pharmacol. 162 (6), 1239–1249. 10.1111/j.1476-5381.2010.01127.x 21091654 PMC3058157

[B29] Irwin J. J. SterlingMysinger T. M. Bolstad E. S. Coleman R. G. Bolstad E. S. Coleman R. G. (2012). Zinc: A free tool to discover chemistry for biology. J. Chem. Inf. Model. 52 (7), 1757–1768. 10.1021/ci3001277 22587354 PMC3402020

[B30] Jura N. Endres N. F. Engel K. Deindl S. Das R. Lamers M. H. (2009). Mechanism for activation of the EGF receptor catalytic domain by the juxtamembrane segment. Cell. 137 (7), 1293–1307. 10.1016/J.CELL.2009.04.025 19563760 PMC2814540

[B31] Kim A. Jang M. H. Lee S. J. Young Bae K. (2017). Mutations of the epidermal growth factor receptor gene in triple-negative breast cancer. J. Breast Cancer 20 (2), 150–159. 10.4048/JBC.2017.20.2.150 28690651 PMC5500398

[B32] Koes D. R. Camacho C. J. (2012). ZINCPharmer: Pharmacophore search of the ZINC database. Nucleic Acids Res. 40 (W1), W409–W414. 10.1093/nar/gks378 22553363 PMC3394271

[B33] Krasinskas A. M. (2011). EGFR signaling in colorectal carcinoma. Pathol. Res. Int. 2011, 932932–932936. 10.4061/2011/932932 PMC3042643 21403829

[B34] Lee S. H. Meng X. W. Flatten K. S. Loegering D. A. Kaufmann S. H. (2013). Phosphatidylserine exposure during apoptosis reflects bidirectional trafficking between plasma membrane and cytoplasm. Cell. Death Differ. 20 (1), 64–76. 10.1038/CDD.2012.93 22858544 PMC3524639

[B35] Li L. Hu M. Wang T. Chen H. Xu L. (2020). Repositioning aspirin to treat lung and breast cancers and overcome acquired resistance to targeted therapy. Front. Oncol. 9, 1503. 10.3389/fonc.2019.01503 31993373 PMC6971167

[B36] Macalino S. J. Y. Gosu V. Hong S. Sun C. (2015). Role of computer-aided drug design in modern drug discovery. Arch. Pharm. Res. 38 (9), 1686–1701. 10.1007/S12272-015-0640-5 26208641

[B37] Martin T. M. Lilavois C. R. Barron M. G. (2017). Prediction of pesticide acute toxicity using two dimensional chemical descriptors and target species classification. Sar. QSAR Environ. Res. 28 (6), 525–539. 10.1080/1062936X.2017.1343204 28703021 PMC5796665

[B38] Martinez-Marti A. Navarro A. Felip E. (2019). Epidermal growth factor receptor first generation tyrosine-kinase inhibitors. Transl. Lung Cancer Res. 8 (3), S235–S246. 10.21037/TLCR.2019.04.20 31857948 PMC6894987

[B39] Masuda H. Zhang D. Chandra B. Doihara H. Hortobagyi G. N. Ueno N. T. (2012). Role of epidermal growth factor receptor in breast cancer. Breast Cancer Res. Treat. 136 (2), 331–345. 10.1007/S10549-012-2289-9 23073759 PMC3832208

[B40] Meng X. Y. Zhang H. X. Mezei M. Cui M. (2011). Molecular docking: A powerful approach for structure-based drug discovery. Curr. Comput. Aided. Drug Des. 7 (2), 146–157. 10.2174/157340911795677602 21534921 PMC3151162

[B41] Morin M. J. (2000). From oncogene to drug: Development of small molecule tyrosine kinase inhibitors as anti-tumor and anti-angiogenic agents. Oncogene 19 (56), 6574–6583. 10.1038/SJ.ONC.1204102 11426642

[B42] Mysinger M. M. Michael Carchia J. J. I. Shoichet B. K. (2012). Directory of useful decoys, enhanced (DUD-E): Better ligands and decoys for better benchmarking. J. Med. Chem. 55 (14), 6582–6594. 10.1021/jm300687e 22716043 PMC3405771

[B43] Nisha C. M. Kumar A. Nair P. Gupta N. Silakari C. Tripathi T. (2016). Molecular docking and in silico ADMET study reveals acylguanidine 7a as a potential inhibitor of β-secretase. Adv. Bioinforma. 2016, 9258578. 10.1155/2016/9258578 PMC4842033 27190510

[B44] Nishikawa S. Kimura H. Koba H. Yoneda T. Watanabe S. Sakai T. (2018). Selective gene amplification to detect the T790M mutation in plasma from patients with advanced non-small cell lung cancer (NSCLC) who have developed epidermal growth factor receptor tyrosine kinase inhibitor (EGFR-TKI) resistance. J. Thorac. Dis. 10 (3), 1431–1439. 10.21037/JTD.2018.01.144 29707292 PMC5906242

[B45] Normanno N. De Luca A. Maiello M. R. Campiglio M. Napolitano M. Mancino M. (2006). The MEK/MAPK pathway is involved in the resistance of breast cancer cells to the EGFR tyrosine kinase inhibitor gefitinib. J. Cell. Physiol. 207 (2), 420–427. 10.1002/JCP.20588 16419029

[B46] Opo F. A. Dain M. Rahman M. M. Ahammad F. Ahmed I. Ahmed Bhuiyan M. (2021). Structure based pharmacophore modeling, virtual screening, molecular docking and ADMET approaches for identification of natural anti-cancer agents targeting XIAP protein. Sci. Rep. 11 (1), 4049. 10.1038/s41598-021-83626-x 33603068 PMC7892887

[B47] Pabla B. Bissonnette M. Konda V. J. (2015). Colon cancer and the epidermal growth factor receptor: Current treatment paradigms, the importance of diet, and the role of chemoprevention. World J. Clin. Oncol. 6 (5), 133–141. 10.5306/WJCO.V6.I5.133 26468449 PMC4600187

[B48] Pao W. Miller V. A. Politi K. A. Riely G. J. Somwar R. Zakowski M. F. (2005). Acquired resistance of lung adenocarcinomas to gefitinib or erlotinib is associated with a second mutation in the EGFR kinase domain. PLoS Med. 2 (3), e73–e35. 10.1371/JOURNAL.PMED.0020073 15737014 PMC549606

[B49] Pokhrel S. Bouback T. A. Samad A. Nur S. M. Alam R. Abdullah-Al-Mamun M. (2021). Spike protein recognizer receptor ACE2 targeted identification of potential natural antiviral drug candidates against SARS-CoV-2. Int. J. Biol. Macromol. 191, 1114–1125. 10.1016/J.IJBIOMAC.2021.09.146 34592225 PMC8474879

[B50] Rahman M. M. Opo F. A. D. M. Asiri A. M. (2021). Cytotoxicity study of cadmium-selenium quantum dots (cdse QDs) for destroying the human HepG2 liver cancer cell. J. Biomed. Nanotechnol. 17 (11), 2153–2164. 10.1166/JBN.2021.3181 34906276

[B51] Rahman M. M. Opo F. A. D. M. Asiri A. M. Rahman M. M. Opo F. A. D. M. AbdullahAsiri M. (2022). Comprehensive studies of different cancer diseases among less-developed countries. Healthcare 10 (3), 424. 10.3390/HEALTHCARE10030424 35326902 PMC8949682

[B52] Saghir S. A. Ahmad Ansari R. (2018). “Pharmacokinetics,” in Reference module in biomedical sciences. NSU Florida, Faculty Books and Book Chapters. 3. 10.1016/B978-0-12-801238-3.62154-2

[B53] Sangande F. Julianti E. Tjahjono D. H. (2020). Ligand-based pharmacophore modeling, molecular docking, and molecular dynamic studies of dual tyrosine kinase inhibitor of EGFR and VEGFR2. Int. J. Mol. Sci. 21 (20), 7779. 10.3390/IJMS21207779 33096664 PMC7590020

[B54] Shi K. Wang G. Pei J. Zhang J. Wang J. Ouyang L. (2022). Emerging strategies to overcome resistance to third-generation EGFR inhibitors. J. Hematol. Oncol. 15 (1), 94. 10.1186/S13045-022-01311-6 35840984 PMC9287895

[B55] Sigismund S. Avanzato D. Lanzetti L. (2018). Emerging functions of the EGFR in cancer. Mol. Oncol. 12 (1), 3–20. 10.1002/1878-0261.12155 29124875 PMC5748484

[B56] Song C. M. ShenLim J. Tong J. C. (2009). Recent advances in computer-aided drug design. Brief. Bioinform. 10 (5), 579–591. 10.1093/BIB/BBP023 19433475

[B57] Spano J. P. Fagard R. Soria J. C. Rixe O. Khayat D. Milano G. (2005). Epidermal growth factor receptor signaling in colorectal cancer: Preclinical data and therapeutic perspectives. Ann. Oncol. 16 (2), 189–194. 10.1093/ANNONC/MDI057 15668269

[B58] Thomas R. Zhang W. (2019). Rethink of EGFR in cancer with its kinase independent function on board. Front. Oncol. 9 (AUG), 800. 10.3389/FONC.2019.00800 31508364 PMC6716122

[B59] Trott O. Olson A. J. (2010). AutoDock Vina: Improving the speed and accuracy of docking with a new scoring function, efficient optimization and multithreading. J. Comput. Chem. 31 (2), 455–461. 10.1002/JCC.21334 19499576 PMC3041641

[B60] Valasani K. R. Vangavaragu J. R. Day V. W. Yan S. S. (2014). Structure based design, synthesis, pharmacophore modeling, virtual screening, and molecular docking studies for identification of novel cyclophilin D inhibitors. J. Chem. Inf. Model. 54 (3), 902–912. 10.1021/ci5000196 24555519 PMC3985759

[B61] Wang Y. Bolton E. Dracheva S. Karapetyan K. Shoemaker B. A. Suzek T. O. (2010). An overview of the PubChem BioAssay resource. Nucleic Acids Res. 38 (1), D255–D266. 10.1093/nar/gkp965 19933261 PMC2808922

[B62] Weng C. W. Wei C. H. Tsai J. Y. Lai Y. H. Chang G. C. JeremyChen J. W. (2022). Hybrid pharmacophore- and structure-based virtual screening pipeline to identify novel EGFR inhibitors that suppress non-small cell lung cancer cell growth. Int. J. Mol. Sci. 23 (7), 3487. 10.3390/IJMS23073487 35408854 PMC8999148

[B63] Wolber G. Langer T. (2005). LigandScout: 3-D pharmacophores derived from protein-bound ligands and their use as virtual screening filters. J. Chem. Inf. Model. 45 (1), 160–169. 10.1021/ci049885e 15667141

[B64] Wykosky J. Fenton T. Frank F. Webster Cavenee K. (2011). Therapeutic targeting of epidermal growth factor receptor in human cancer: Successes and limitations. Chin. J. Cancer 30 (1), 5–12. 10.5732/CJC.010.10542 21192840 PMC3359794

[B65] Yadav S. Pandey S. K. Singh V. K. Goel Y. Kumar A. Singh S. M. (2017). Molecular docking studies of 3-bromopyruvate and its derivatives to metabolic regulatory enzymes: Implication in designing of novel anticancer therapeutic strategies. PloS One 12 (5), e0176403. 10.1371/JOURNAL.PONE.0176403 28463978 PMC5413015

[B66] Yan X. E. Ayaz P. Zhu S. J. Zhao P. Liang L. Zhang C. H. (2020). Structural basis of AZD9291 selectivity for EGFR T790M. J. Med. Chem. 63 (15), 8502–8511. 10.1021/ACS.JMEDCHEM.0C00891 32672461

[B67] Yoon H-Y. Ryu J-S. Su Sim Y. Kim D. Sung Lee Y. Choi J. (2020). Clinical significance of EGFR mutation types in lung adenocarcinoma: A multi-centre Korean study. PLOS ONE 15 (2), e0228925. 10.1371/JOURNAL.PONE.0228925 32053675 PMC7018076

[B68] Yun C-H. Boggon T. J. Li Y. Woo M. S. Greulich H. Meyerson M. (2007). Structures of lung cancer-derived EGFR mutants and inhibitor complexes: Mechanism of activation and insights into differential inhibitor sensitivity. Cancer Cell. 11 (3), 217–227. 10.1016/J.CCR.2006.12.017 17349580 PMC1939942

[B69] Yun C. H. Mengwasser K. E. Toms A. V. Woo M. S. Greulich H. Kwok Wong K. (2008). The T790M mutation in EGFR kinase causes drug resistance by increasing the affinity for ATP. Proc. Natl. Acad. Sci. U. S. A. 105 (6), 2070–2075. 10.1073/PNAS.0709662105 18227510 PMC2538882

[B70] Yunta M. J. R. (2017). It is important to compute intramolecular hydrogen bonding in drug design? Am. J. Model. Optim. 5 (1), 24–57. 10.12691/AJMO-5-1-3

[B71] Zhang X. Zhang B. Liu J. Liu J. Li C. Dong W. (2015). Mechanisms of gefitinib-mediated reversal of tamoxifen resistance in MCF-7 breast cancer cells by inducing ERα Re-expression. Sci. Rep. 5 (1), 7835–7837. 10.1038/srep07835 25644501 PMC4314651

