# Supplementary material for: Pharmacophore-based virtual screening approaches to identify novel molecular candidates against EGFR through comprehensive computational approaches and in-vitro studies
Source: Front Pharmacol. 2022 Nov 15;13:1027890. doi: 10.3389/fphar.2022.1027890 (PMC9707641; doi:10.3389/fphar.2022.1027890)
Supplement: Supplementary file 1 [file DataSheet1.PDF]

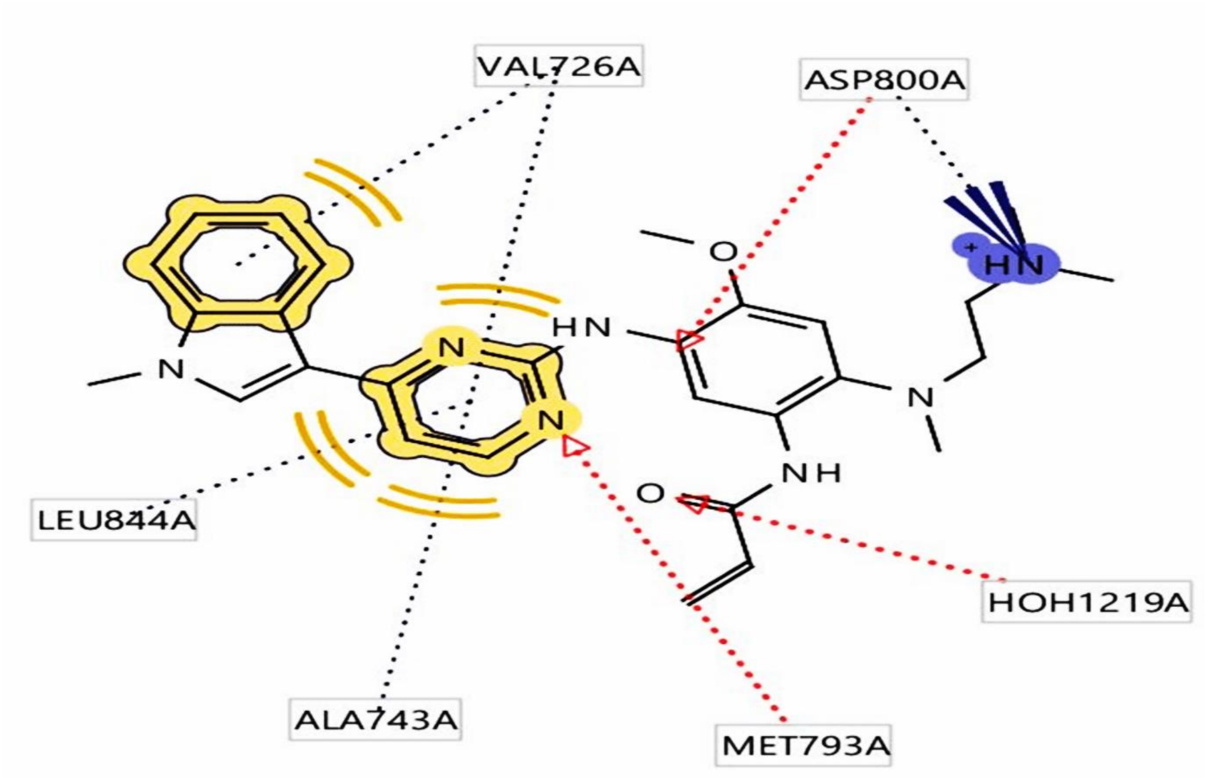

Figure S1: The 2D-depiction generated from Ligand Scout 4.4.8 advance software, yellow color showing hydrophobic pocket. Hydrophobic type of interactions is mostly predominant in the 2D depiction model, indicated by black color. Red color showing the hydrogen bond acceptor features with several functional groups.

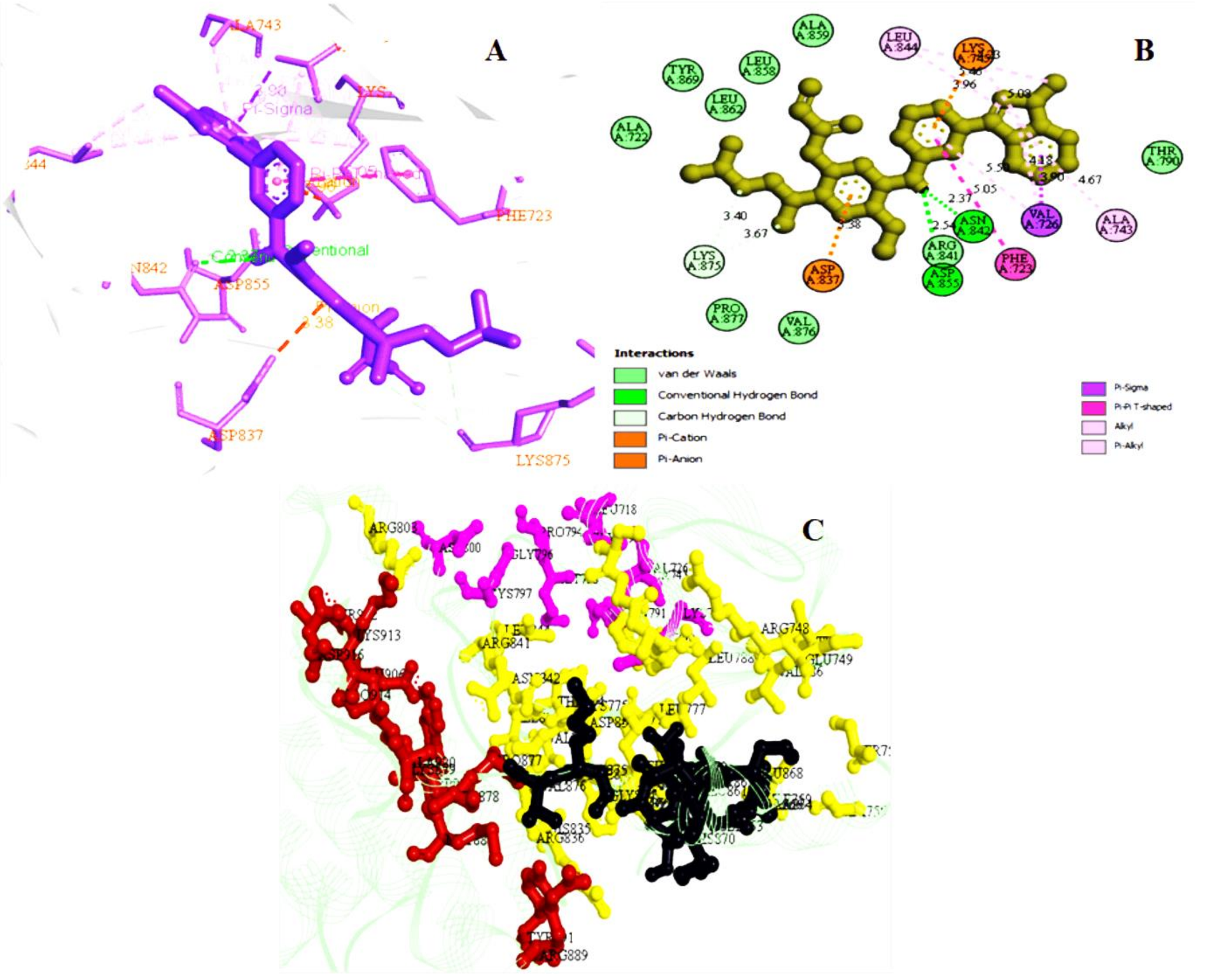

Figure S2: Describing protein ligand interaction generated from protein ligand interaction complex and the active site of the EGFR. (A) 3D Interaction of the protein ligand complex (B) Bond formation with attached ligand (C) the number of attached active site with 6JXT protein.

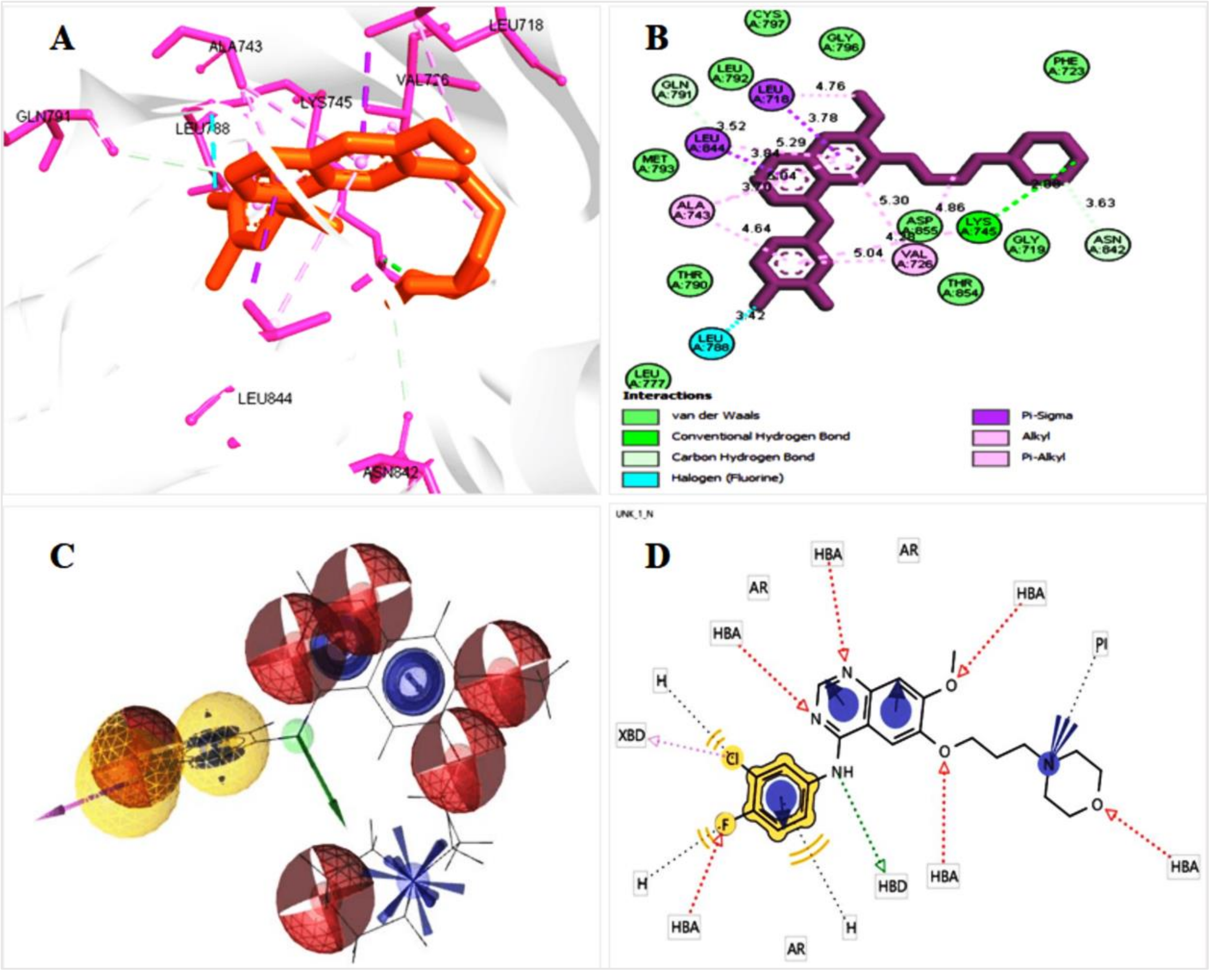

Figure S3: EGFR tyrosine kinase inhibitor (Gefitinib) with the 3D, 2D interaction, obtained pharmacophorre features 3Dand 2D. Gefitinib was docked with EGFR protein and generated all the features.

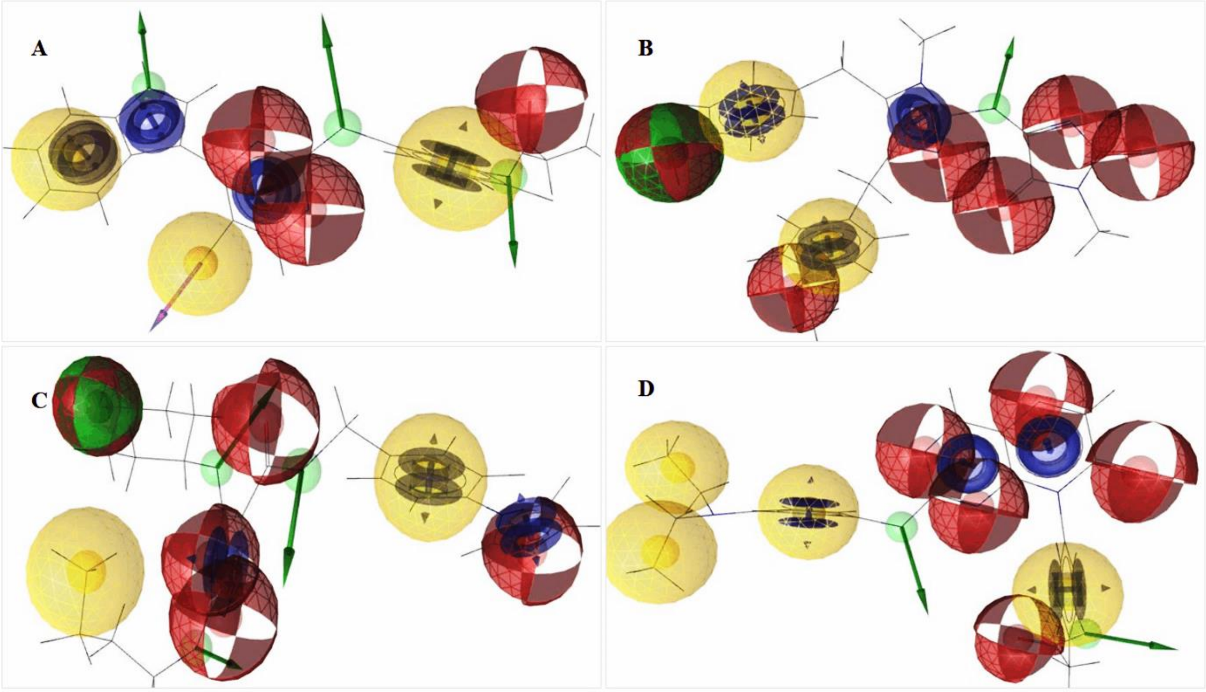

Figure S4: Several pharmacophore features of our selected compounds and ligand attach to EGFR protein. Selected protein attached to the ligand were indicated by pharmacophore features. Our natural compounds were showing more pharmacophore features and the binding capacity was higher with following the Lipinski rule of five (R05).

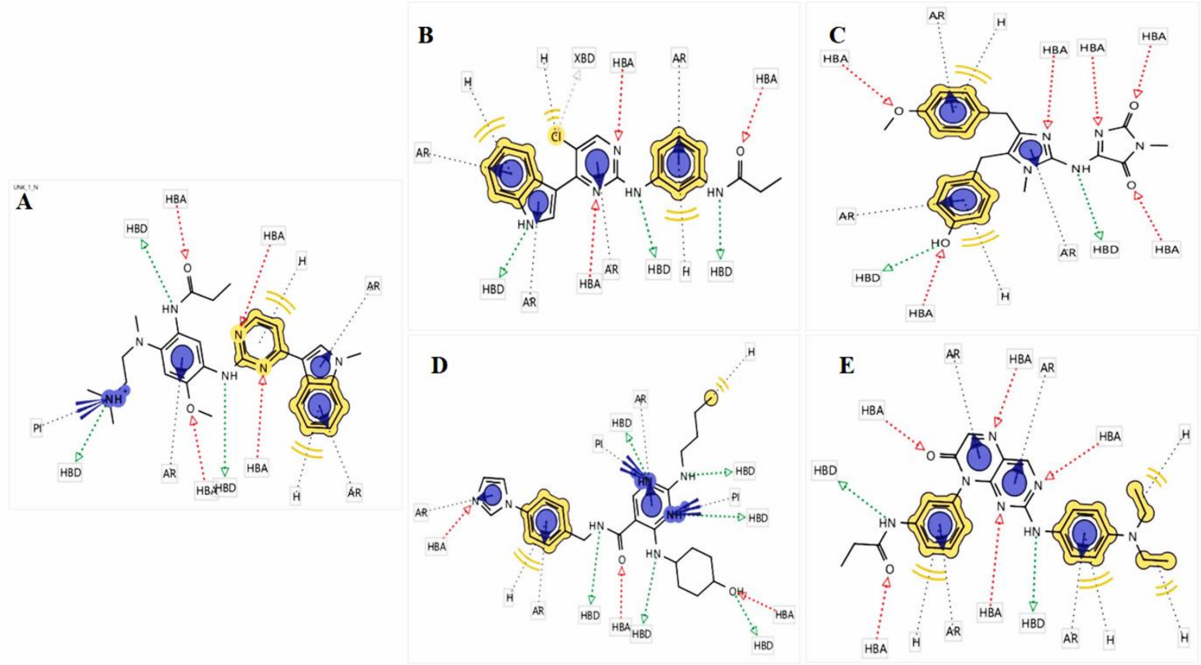

Figure S5: Analysis of 2d pharmacophore features of our selected antagonists and also control ligand. The result revealed that our compounds had more features than the current ligand attached to the selected protein and fowling the Lipinski rule of five.

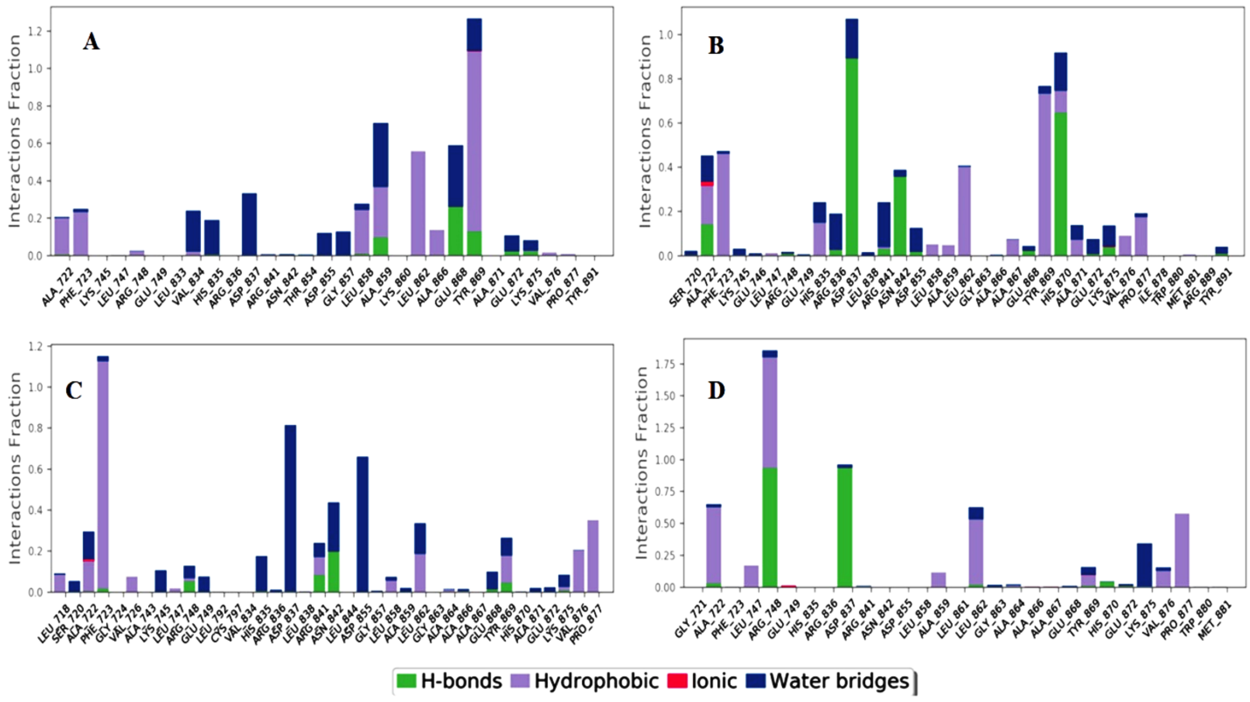

Figure S6: The column graph indicated the protein-ligand interaction during the molecular dynamic simulation at 100ns. The graph was showed the bond contact with several amino acids residue.

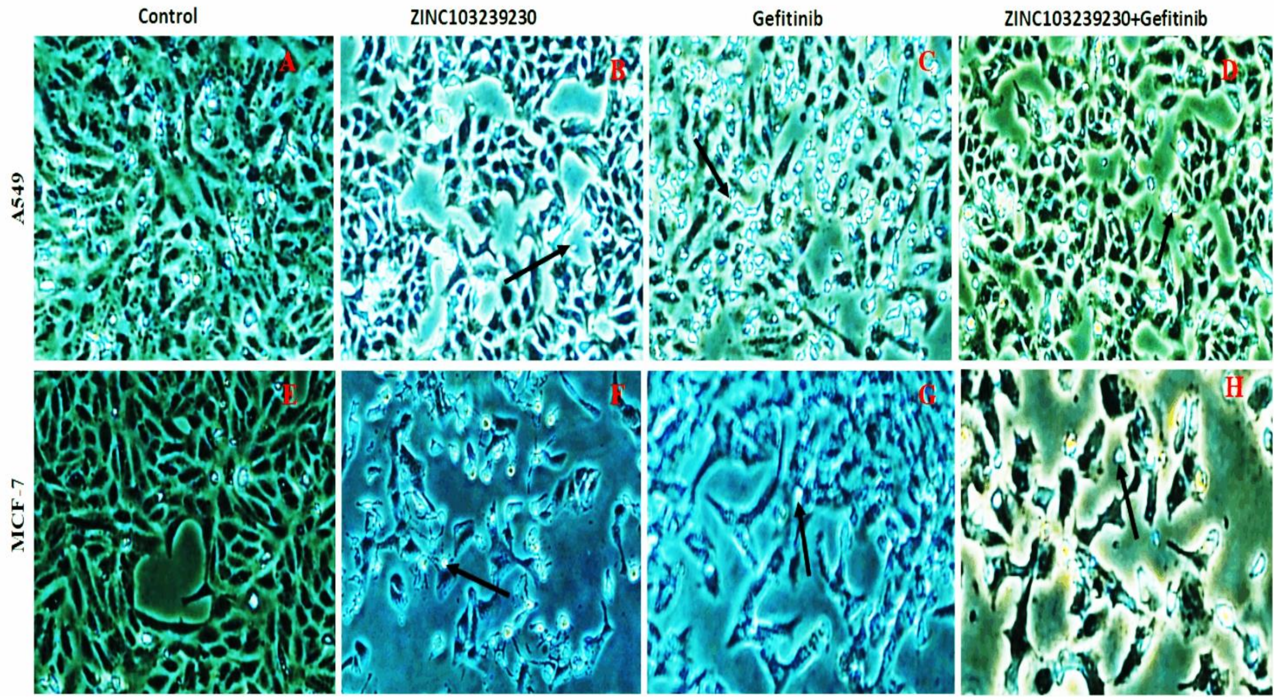

Figure S7: The cell death pattern through using different drugs with IC_{50} concentration. (A) A549 cell without the treatment. (B, C, D) indicated the cell death by their structural changes (Elongation, cell shrinkage, cell membrane rupture). MCF-7 cell death indicated by F, G, H and E showed the MCF-7 cell line without treatment. Black arrows indicating the death cell. All photos were taken by phase contrast microscope (Scale bar 100µm)

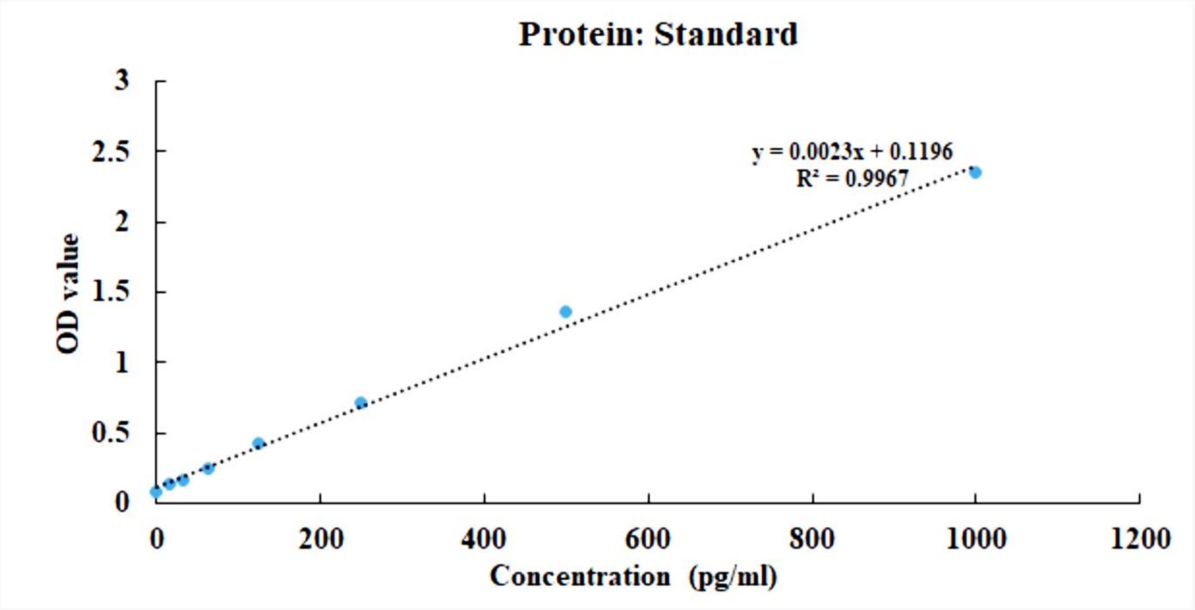

Figure S8: The standard curve preparation for the EGFR protein.

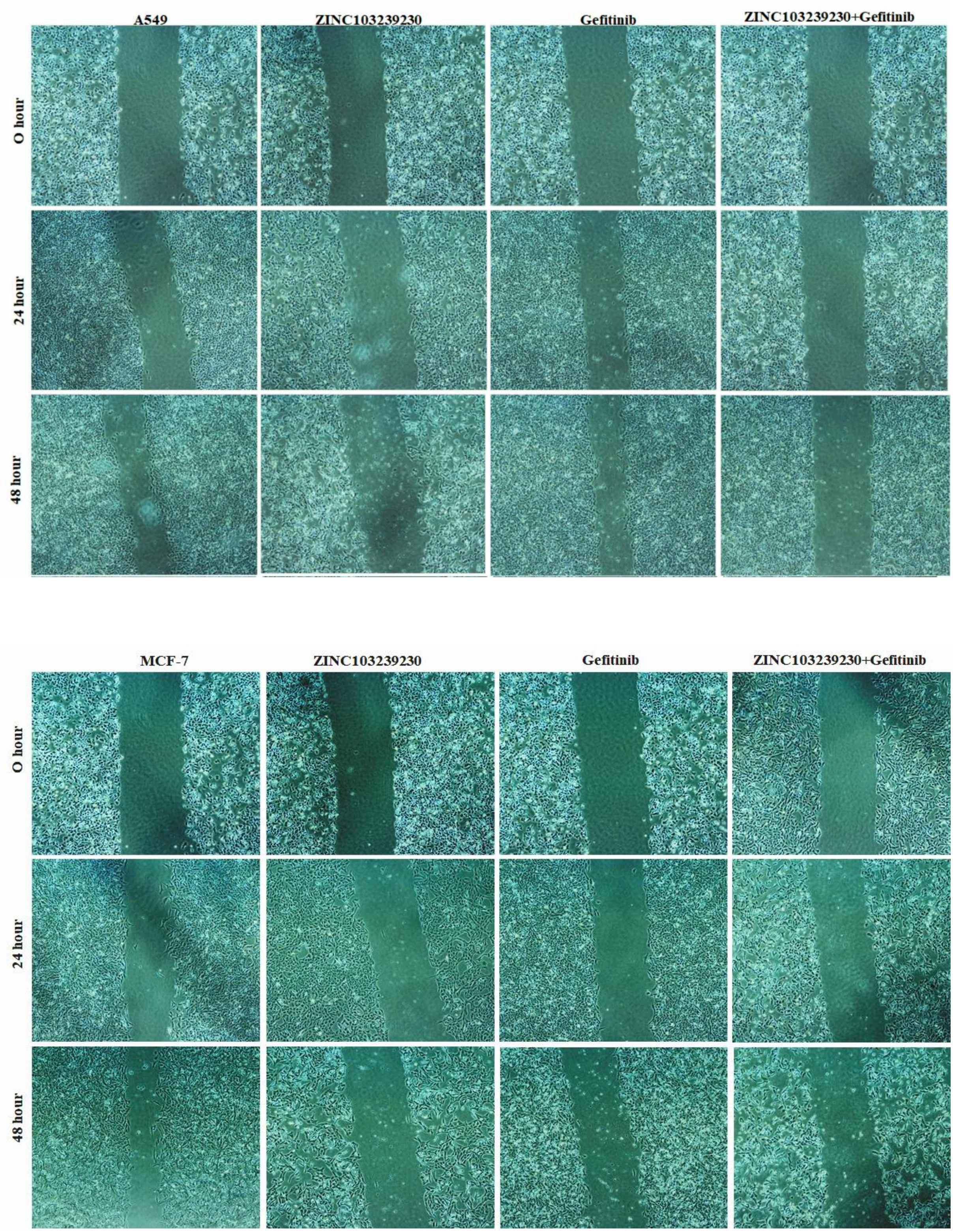

Figure S9: The migration of the cell with presence of antagonist and marketed drug gefitinib. A549 column were used as a control and was considered as untreated, at the same time MCF-7 column indicated control group without treatment.

Table S1: The purchased of ZINC103239230 from the S Molecule. The gefitinib also purchased from the local pharmaceutical company of Bangladesh

**mcule.com Kft.**		**Quotation reference**	Q-11505
Bartók Béla út 105-113.		**Customer**	Dain Md. Opu
H-1115, Budapest		**Quotation date**	July 21, 2021
Hungary		**Valid until**	August 20, 2021
Tax ID: HU23383540		**Payment terms**	30 days net or prepayment by credit card
		**Estimated delivery time**	14 working days after purchase order accepted
Email: order@mcule.com		**Delivery format**	Dry powder/film in supplier vial
Phone: +36-1-781-5983		**Shipping country**	Saudi Arabia
**Quoted Mcule ID**		**Quoted product SMILES**	
MCULE-3322057278		CCCCNC1N=C(NC2CCC(O)CC2)C(C(NCC2C=CC(N3C=NC=C3)=CC=2)=O)=CN=1	
**Item**	**Number of products**	**Amount / product (mg)**	**Average product price (USD)**
Products	1	5	197.02
Shipping (from supplier to Mcule)			139.57
Shipping & handling			44.07
**Total**			**380. 66**
New customers are eligible for 10% discount if indicated at the time of ordering (will be deduced from your first invoice).			

Table S2: The number of selected known inhibitor with their IC_{50} value, molecular formula and chemical structure

Number	PubChem ID	Molecular formula	Chemical Structure	IC50(µM)
1	176870	$C_{22}H_{23}N_3O_4$	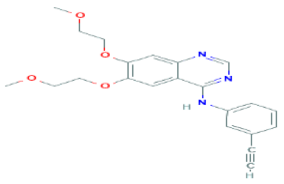	0.0001
2	375472	$C_{14}H_{11}NO_5$	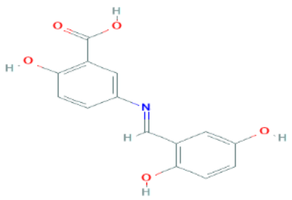	5

3	5328837	$C_{20}H_{19}BrN_2O_4$	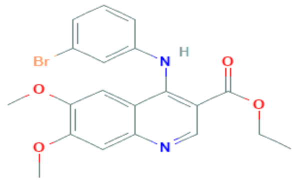	6.94
4	5328863	$C_{21}H_{21}N_3O_2$	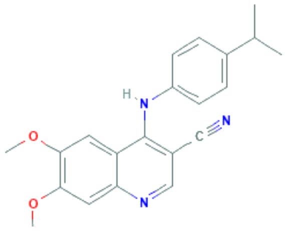	0.23
5	6438552	$C_{15}H_{24}O_7$	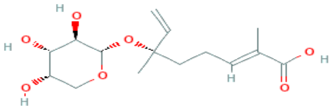	9.3
6	9859850	$C_{14}H_{14}N_4O$	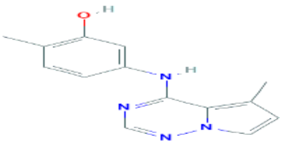	0.34
7	10199254	$C_{15}H_{16}N_4O$	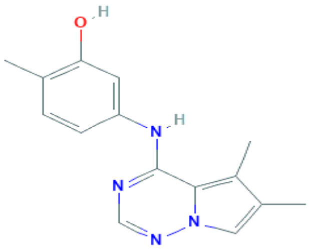	0.2
8	10499929	$C_{21}H_{21}N_3O_4$	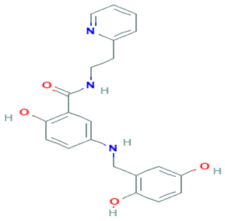	0.015
9	11357877	$C_{13}H_{10}ClFN_4$	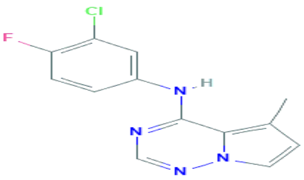	0.1

10	44307008	$C_{18}H_{17}NO_7$	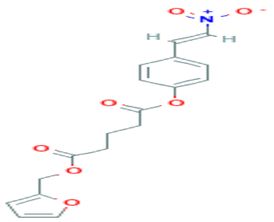	0.29
11	44345316	$C_{17}H_{15}N_3O_3S$	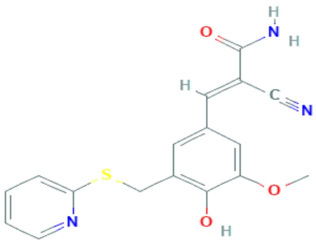	3.38844
12	44345633	$C_{19}H_{17}ClN_2O_3S$	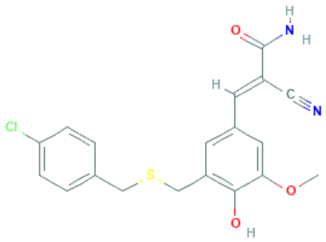	3
13	14311151	$C_{22}H_{23}O_{11}^{+}$	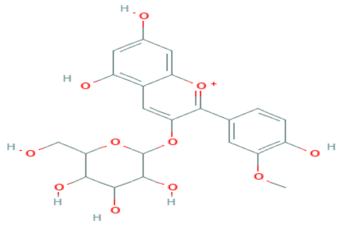	0.50
14	135511071	$C_{23}H_{23}N_5O_4$	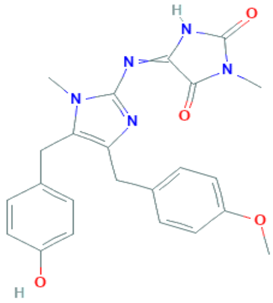	9.53
15	44372197	$C_{27}H_{25}N_5O_3S_2$	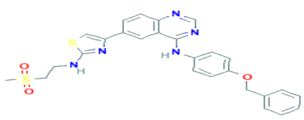	0.082
16	44372283	$C_{28}H_{24}F_3N_5O_3S_2$	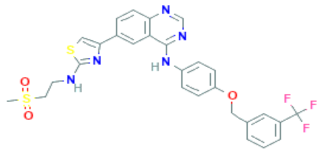	0.54

17	49860603	$C_{20}H_{21}ClN_4O_2$	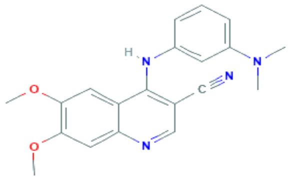	0.85
18	71721525	$C_{25}H_{33}N_7O_2$	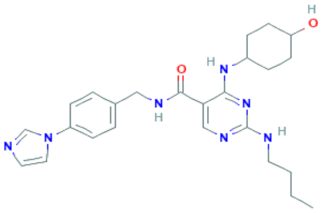	0.32
19	72202868	$C_{25}H_{25}N_7O_2$	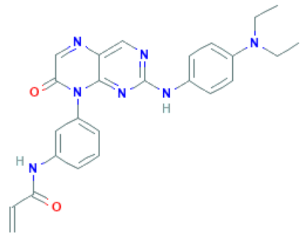	0.00472
20	73294313	$C_{25}H_{25}ClN_6O_2$	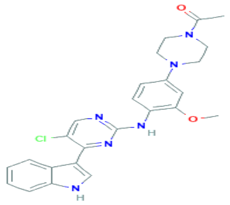	0.77
21	73294314	$C_{21}H_{16}ClN_5O$	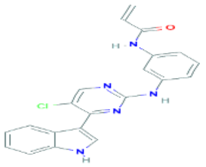	0.81
22	85471121	$C_{28}H_{26}ClF_2N_5O_3$	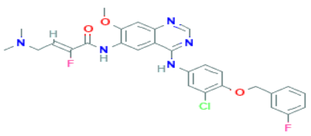	0.00156
23	118706214	$C_{19}H_{17}N_5O_3$	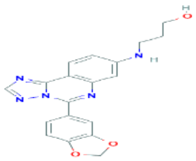	2.56

24	127050068	$C_{28}H_{25}ClFN_5O_5$	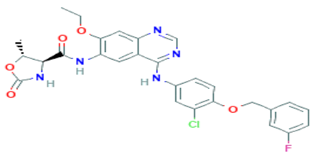	0.00257
25	127052091	$C_{21}H_{22}ClFN_6OS$	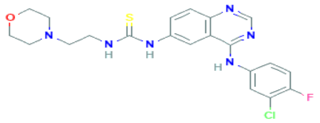	0.15
26	76900441	$C_{25}H_{20}ClN_3O_3$	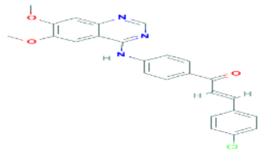	0.027
27	11566580	$C_{17}H_{11}ClFN_5O$	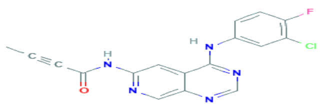	0.0025
28	127264586	$C_{25}H_{24}ClN_7O_2$	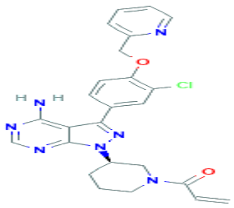	0.0001
29	121280087	$C_{30}H_{35}N_7O_2$	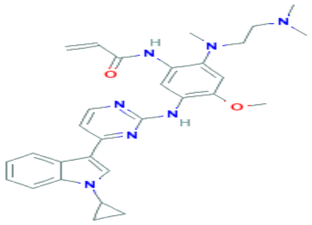	0.53
30	89670174	$C_{25}H_{17}F_3N_4O_3$	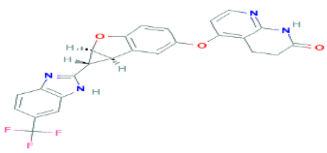	0.029
31	72734520	$C_{26}H_{26}FN_7O_2$	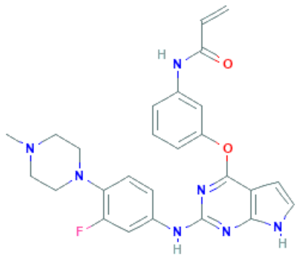	0.18

32	123631	$C_{22}H_{24}ClFN_4O_3$	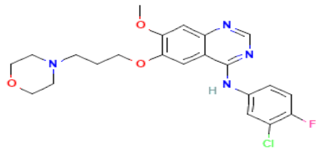	2.4

Table S3: Types of bond formed among the selected antagonist with their distance, category and the bond formation residue.

Zinc ID	Residue	Distance	Category	Type
ZINC96937394	MET793	2.6294	Hydrogen Bond	Conventional Hydrogen Bond
	ASP855	3.4444	Electrostatic	Pi-Anion
	LEU718	3.83771	Hydrophobic	Pi-Sigma
	THR790	3.68495	Hydrophobic	Pi-Sigma
	ALA743	5.13206	Hydrophobic	Pi-Alkyl
	LEU844	4.95436	Hydrophobic	Pi-Alkyl
	LYS745	4.81269	Hydrophobic	Pi-Alkyl
	LEU788	5.16262	Hydrophobic	Pi-Alkyl
	ALA743	4.70503	Hydrophobic	Pi-Alkyl
	LEU844	4.44865	Hydrophobic	Pi-Alkyl
	LEU718	5.18615	Hydrophobic	Pi-Alkyl
	MET793	1.61032	Unfavorable	Unfavorable Donor-Donor
ZINC14611940	LYS745	3.07377	Hydrogen Bond	Conventional Hydrogen Bond
	LYS745	2.80168	Hydrogen Bond	Conventional Hydrogen Bond
	THR790	2.29469	Hydrogen Bond	Conventional Hydrogen Bond
	LEU718	3.71047	Hydrogen Bond	Carbon Hydrogen Bond
	LEU788	3.47693	Hydrogen Bond	Carbon Hydrogen Bond
	ASP855	4.25467	Electrostatic	Pi-Anion
	LEU788	5.31726	Hydrophobic	Alkyl
	LYS745	3.86388	Hydrophobic	Alkyl
	LEU718	4.8807	Hydrophobic	Pi-Alkyl
	ALA743	5.01775	Hydrophobic	Pi-Alkyl
	LEU844	4.60319	Hydrophobic	Pi-Alkyl
	VAL726	4.51917	Hydrophobic	Pi-Alkyl
	ALA743	4.81401	Hydrophobic	Pi-Alkyl
	LYS745	5.44437	Hydrophobic	Pi-Alkyl
	MET793	1.06919	Unfavorable	Unfavorable Donor-Donor
ZINC103239230	ARG841	2.34138	Hydrogen Bond	Conventional Hydrogen Bond
	MET793	2.21793	Hydrogen Bond	Conventional Hydrogen Bond
	GLN438	2.68776	Hydrogen Bond	Conventional Hydrogen Bond
	THR503	2.77995	Hydrogen Bond	Conventional Hydrogen Bond
	ASP855	3.42001	Hydrogen Bond	Carbon Hydrogen Bond
	MET766	3.47288	Hydrogen Bond	Carbon Hydrogen Bond
	ASP855	3.72526	Electrostatic	Pi-Anion
	THR790	3.24363	Hydrophobic	Pi-Sigma
	LEU718	4.8546	Hydrophobic	Alkyl
	ALA743	4.67276	Hydrophobic	Alkyl
	LEU844	4.70735	Hydrophobic	Alkyl

	VAL726	4.71853	Hydrophobic	Pi-Alkyl
	MET766	4.40067	Hydrophobic	Pi-Alkyl
ZINC96933670	ARG841	2.82416	Hydrogen Bond	Conventional Hydrogen Bond
	TYR869	2.80397	Hydrogen Bond	Conventional Hydrogen Bond
	GLU868	3.5239	Hydrogen Bond	Carbon Hydrogen Bond
	ASP837	3.5371	Electrostatic	Pi-Anion
	PHE723	5.93153	Hydrophobic	Pi-Pi T-shaped
	ALA722	4.75293	Hydrophobic	Pi-Alkyl
	ALA722	4.24809	Hydrophobic	Pi-Alkyl
	ALA859	5.45543	Hydrophobic	Pi-Alkyl
